# A bioengineered artificial interstitium supports long-term islet xenograft survival in nonhuman primates without immunosuppression

**DOI:** 10.1126/sciadv.adi4919

**Published:** 2024-01-05

**Authors:** Scott H. Oppler, Laura L. Hocum Stone, David J. Leishman, Jody L. Janecek, Meghan E. G. Moore, Parthasarathy Rangarajan, Bradley J. Willenberg, Timothy D. O’Brien, Jaime Modiano, Natan Pheil, Jordan Dalton, Michael Dalton, Sabarinathan Ramachandran, Melanie L. Graham

**Affiliations:** ^1^ Department of Surgery, University of Minnesota, Minneapolis, MN, USA.; ^2^ Department of Veterinary Clinical Sciences, College of Veterinary Medicine, University of Minnesota, St. Paul, MN, USA.; ^3^ Department of Internal Medicine, University of Central Florida College of Medicine, Orlando, FL, USA.; ^4^ Department of Veterinary Population Medicine, University of Minnesota, St. Paul, MN, USA.; ^5^ Cell-Safe LifeSciences, Skokie, IL, USA.; ^6^ Medline UNITE Foot and Ankle, Medline Industries LP, 3 Lakes Drive, Northfield, IL, USA.

## Abstract

Cell-based therapies hold promise for many chronic conditions; however, the continued need for immunosuppression along with challenges in replacing cells to improve durability or retrieving cells for safety are major obstacles. We subcutaneously implanted a device engineered to exploit the innate transcapillary hydrostatic and colloid osmotic pressure generating ultrafiltrate to mimic interstitium. Long-term stable accumulation of ultrafiltrate was achieved in both rodents and nonhuman primates (NHPs) that was chemically similar to serum and achieved capillary blood oxygen concentration. The majority of adult pig islet grafts transplanted in non-immunosuppressed NHPs resulted in xenograft survival >100 days. Stable cytokine levels, normal neutrophil to lymphocyte ratio, and a lack of immune cell infiltration demonstrated successful immunoprotection and averted typical systemic changes related to xenograft transplant, especially inflammation. This approach eliminates the need for immunosuppression and permits percutaneous access for loading, reloading, biopsy, and recovery to de-risk the use of “unlimited” xenogeneic cell sources to realize widespread clinical translation of cell-based therapies.

## Teaser

Bioartificial interstitium for diabetes therapy eliminates immunosuppression in islet transplant using unlimited xenogenic cells.

## INTRODUCTION

Cell-based therapy spans multiple therapeutic areas for treatment of chronic disease conditions including endocrine disorders, neurodegenerative disorders, cardiovascular disease, cancer, and autoimmune disorders (*1*–*8*). Cell-based therapies sense pathologic changes in the physiologic state to deliver therapeutic molecules that can include enzymes, hormones, mRNA, and neuromodulators, a unique mode of action unlike most conventional treatment modalities (*2*, *3*, *5*, *8*–*10*). Despite the notable successes, cell-based therapies still face a number of challenges, primarily related to safe and effective cell delivery and durability of the therapy. As with solid organ transplantation, therapeutic cells may be immunogenic to the recipient and therefore require chronic immunosuppression which can result in a range of side effects and increase risk of infection (*3*, *11*–*16*). To this end, various cell encapsulation techniques using semipermeable barriers to immuno-isolate cells have offered promise for achieving these goals in the absence of immunosuppression (*17*, *18*). However, the design of encapsulation technology requires careful consideration of a number of factors to create an optimal biomimetic environment suitable for cell survival that also addresses the critical needs of clinical therapy, including biocompatibility, immune protection, biomimetic vascularization, prevention of hypoxia-induced cell loss, easy access to device for cell replacement or retrieval, and the promotion of patient compliance (*19*, *20*), factors that have been persistent problems over time with conventional encapsulation technologies (*19*).

Allogeneic islet cell transplant for type I diabetes mellitus (T1DM) is a prime example of the actualization of a cell-based alternative to conventional chronic insulin therapy, as it has been demonstrated that β cell replacement results in near normoglycemia (*21*), reduces risk of hypoglycemic attacks, and improves or slows progression of microvascular complications by providing a steady-state physiologic source of insulin (*22*–*27*). The prevalence of T1DM is estimated to be about 10% of the US population (*28*) with incidence and prevalence continuing to increase both nationally and globally (*29*). Patients with longstanding T1DM suffer from heightened morbidity associated with development of micro- and macrovascular disease, including retinopathy (*30*, *31*), neuropathies (*32*, *33*), nephropathy (*34*), and potentially life-threatening hypoglycemic events associated with intensive insulin therapy (*35*–*38*). Current standard of care for T1DM requires routine blood glucose monitoring and exogenous insulin administration, a nonphysiologic treatment resulting in the development of secondary complications and, consequently, reduced quality-of-life and increased burden of disease (*30*, *31*, *35*–*46*). Successful β cell replacement therapy can minimize these complications while also reducing the patient’s burden of care introduced by more typical intensive therapy.

Despite the success of islet cell transplantation, there are obstacles remaining related to sourcing sufficient islets, instant blood-mediated inflammatory reaction (IBMIR) damaging cells at delivery, chronic immunosuppression, and loss of function over time that each hinder long-term functional graft and therapeutic success (*2*, *9*, *13*, *14*, *47*, *48*). Several groups have attempted to overcome the hurdle of cell sourcing by using porcine islets, an unlimited cell source, and have successfully demonstrated that pig islet xenografts can reverse diabetes for prolonged periods in nonhuman primates (NHPs). However, NHPs required either clinically unavailable immunosuppression, relied on islet-toxic regimens, or had safety concerns precluding successful translation (*49*–*53*). Transplanted islets are generally delivered intraportal, where they are immediately subjected to IBMIR, resulting in inflammation, hypoxia, exposure to proinflammatory blood components, complement cascades, and neutrophil infiltration (*54*). Similar reactions can cause an early loss of transplanted cells, complicating dosing and threatening long-term graft function. To address IBMIR and protect the graft from rejection, patients are exposed to immunosuppression, at induction and long-term, along with the associated infectious risks and side effects (*3*, *11*–*16*). Recent clinical trial publications on intraportal infusion of purified human pancreatic islet products have demonstrated long-term efficacy in T1DM (*55*–*57*); however, these protocols still require immunosuppression and invasive abdominal surgery for infusion.

**Fig. 1.**
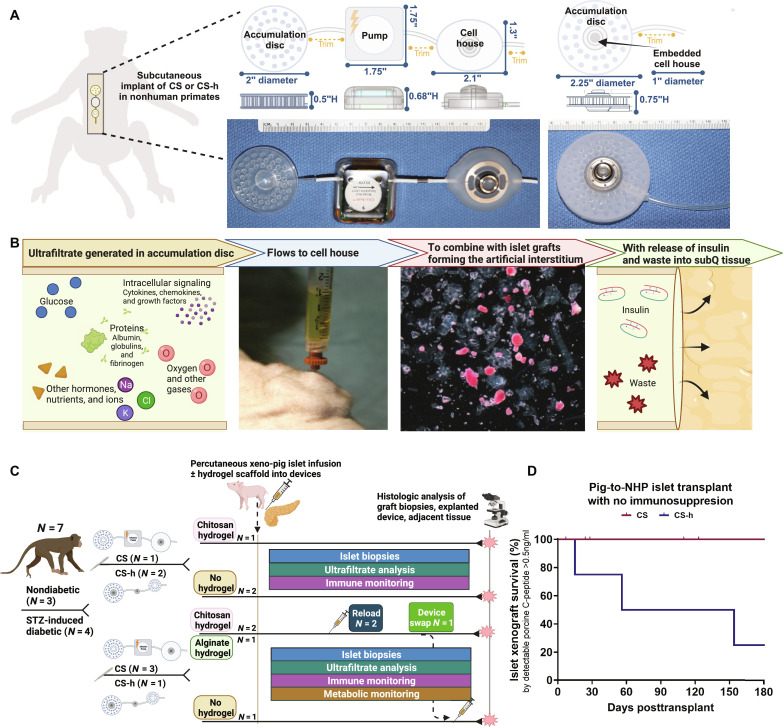
Overview of the CS device. (**A**) The CS and CS-h devices are implanted within the subcutaneous tissues. Middle: Schematic of the three-component CS device which includes a fluid AD, piezoelectric pump to control flow rate, and cell house. Right: Schematic of the CS-h device composed of a fluid AD dependent on hydrostatic pressure gradients with an embedded cell house chamber. (**B**) Flow of UF through the CS device and formation of the artificial interstitium. Left: UF collects in the fluid AD, is mainly deplete of immune cells, and exchanges factors necessary for graft survival and function. Left middle: UF flows via convection, whether by pump or osmotic gradient, into the cell house; the cell house is readily accessible percutaneously to collect UF for characterization, deliver cell constructs, perform biopsies, or retrieve the entire graft. Right middle: UF and the islet-hydrogel constructs combine to form the artificial interstitium within the cell house where the islet grafts can sense and respond to multiple cellular signals including glucose. An inverted micrograph is depicted of purified porcine islets in capillary alginate hydrogel matrix following injection (loading) and aspiration (biopsy) via a 19-gauge needle on the day of transplantation, islets maintained their solid spheroid appearance staining strongly positive with DTZ. Right: The convective flow carries insulin and waste products released by islet grafts into the surrounding subcutaneous tissues via outflow tube. (**C**) Study design overview in NHPs, including health status, device type, and graft composition. (**D**) Kaplan-Meier estimates of rejection-free pig islet xenograft survival in NHPs calculated from the date of transplantation to the date of graft failure as measured by C-peptide (≤0.5 ng/ml). In the event of device removal, retransplantation, or planned termination with a functioning graft, the follow-up period is censored at the date of event.

Our experimental goal was to develop an encapsulation device—in this application to act as a bioartificial pancreas—designed to overcome the major barriers currently present in cell transplantation and encapsulation, including (i) cost, (ii) limited alternative sources of therapeutic cells, (iii) lack of standardized product, (iv) the IBMIR or other immune-mediated threats of cell rejection, (v) cell survival, (vi) major toxicity associated with life-long immunosuppression, and (vii) the ability to retransplant or reload (*58*). The Cell-Safe (CS) and Cell-Safe hybrid (CS-h) percutaneously accessible devices (Fig. 1, A and B) use a unique conceptual approach of convective exchange of autologous blood ultrafiltrate (UF) to support immunoisolated islets and can be combined with an injectable hydrogel designed to provide a physiological three-dimensional (3D) microenvironment that supports cell survival and function. The subcutaneously implanted devices use a tissue engineering strategy to exploit the normal physiologic response where fluid from the vascular space filters into a void, serving as both a transport medium for gas, nutrients, and waste products, as well as signaling proteins and as a natural barrier to protect transplanted cells from immune rejection (Fig. 1B). The resulting microenvironment, when combined within a 3D structured hydrogel located in the device’s cell house, uniquely mimics the interstitial space to support survival and function of transplanted cells. In addition to overcoming the obstacles presented by traditional cell therapy delivery, this approach presents unprecedented opportunity to investigate the influence of factors in the immediate graft environment on cell survival and function via noninvasive cell biopsy or UF collection. Here, we demonstrate that the CS and CS-h devices have the potential to meet the clinical need for safe and effective cell replacement therapy.

## RESULTS

### Device fabrication

The CS device is a three-component device that is fully implantable in the subcutaneous layer designed to provide isolation, nutrition, and oxygen to transplanted cells while also allowing for individual component exchange or total device exchange (Fig. 1A). The components (Fig. 1A and fig. S1) include the (i) accumulation disc (AD), (ii) piezoelectric programmable flow pump, and (iii) cell house (isolation chamber) connected inline by silicone tubing. The AD is made of medical-grade silicone, engineered to create a void or open space in the subcutaneous tissue via a pressure gradient, so that interstitial fluid moves from the surrounding tissue into the disc space where UF accumulates. The pump draws fluid from the disc and pushes it into the cell house. The normal hydrostatic pressure of the subcutaneous space replaces the fluid drawn off via pumping. The cell house had a central 1.25- to 2-ml chamber surrounded by a 30- to 75-μm donut filter or 25- to 35-μm inlet/90- to 150-μm outlet filter. The central chamber is topped with a port that can be accessed percutaneously for noninvasive islet cell or islet cell hydrogel construct loading and reloading, as well as repeated UF analysis and islet graft biopsy or retrieval (Fig. 1B). A representative darkfield image of islet product on the day of transplantation shows intact, functional islets stained with DTZ, that maintain structure following passage through a needle (Fig. 1B). In combination, these components provide transplanted cells with a protected physiologic environment for survival while permitting active fluid transfer of ions, proteins, dissolved gases, nutrient molecules, and wastes (Fig. 1B).

The CS-h (Fig. 1A and fig. S1) is a simplified version of the CS device that relies only on hydrostatic pressure to drive UF exchange within the cell chamber in an effort to mimic the dynamic nature of interstitium in the absence of a mechanical pump component. An outflow tube connects the AD and embedded cell house to distal subcutaneous space, and UF is drained on the basis of the pressure difference between the two cavities to provide continuous drainage. The device action exploits innate mechanisms of vascular permeability, the hydrostatic and plasma colloid osmotic differential, for gas, nutrient, and waste exchange.

The CS and CS-h devices were tested in vitro to evaluate hydrogel suitability in supporting graft survival and function, in rats to test AD biocompatibility for basic safety as well as characterize the stability and persistence of accumulated UF, and then implanted in both healthy and diabetic NHPs to assess device graft survival and function under various conditions (Fig. 1C) that included planned retransplant, which is anticipated as necessary based on the current allograft clinical experience or potential device swap.

### In vitro simulation of planned perfusion conditions and injectable hydrogels on islet cell survival function

**Fig. 2.**
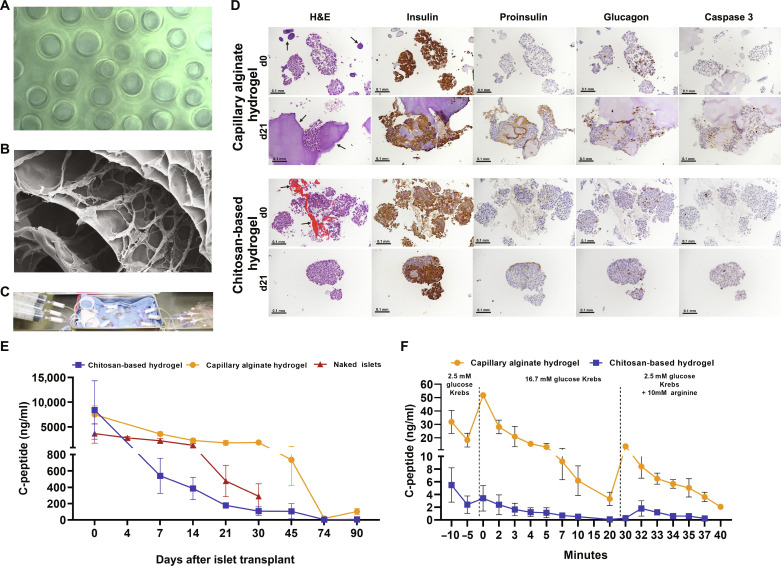
In vitro optimization of islet graft hydrogel support within the CS. (**A**) Phase-contrast image of the capillary alginate injectable hydrogel, showing the structure of patent microcapillary microchannels that run in parallel through the gel. (**B**) Electron micrograph image of the chitosan-based hydrogel. (**C**) In vitro assembled CS device. Islet grafts (120,000 IEQ) in the CS were perfused with culture media using a syringe pump at a constant low volumetric rate of 2 ml/hour anticipated to mimic achievable flow rates in vivo. (**D**) Representative islet graft biopsies with capillary alginate and chitosan-based hydrogel support taken at the day of transplantation (d0) and 21 days following transplantation (d21). Islets in both preparations stain positive for insulin at d21, with minimal staining for proinsulin, glucagon, and cleaved caspase 3 (CC3). Scale bars, 0.1 mm. Representative arrows indicate hydrogel. (**E**) Comparison of hydrogel-supported graft function by C-peptide detection up to 90 days following transplantation (alginate: *n* = 5, chitosan: *n* = 3, naked: *n* = 6). (**F**) GSIS comparison of glucose responsiveness in grafts with capillary alginate and chitosan-based hydrogel support (*n* = 2). Data presented as mean ± SEM. H&E, hematoxylin and eosin.

In vitro studies modeled perfusion with UF using islet-specific culture media perfused at a rate of 2 ml/hour via a Harvard pump (Fig. 2C) at 37°C. Following 7 days conventional static culture, islets were successfully loaded into 32 devices in cell doses ranging from 12,000 to 200,000 islet equivalents (IEQ). Islets were transplanted naked, as well as supported by either a capillary alginate (Fig. 2A) or chitosan-based (Fig. 2B) injectable hydrogel to provide a physiological 3D microenvironment.

Islets exhibited stable insulin production for up to 30 days and remained viable for up to 90 days under perfusion conditions in the in vitro CS device. On the day of implantation (d0), biopsies showed intact, viable islets, with no evidence of apoptosis with islets exhibiting strong intracytoplasmic immunopositivity for insulin and glucagon and no immunoreaction (negative) for proinsulin and cleaved caspase 3 (CC3). Day 21 biopsies showed intact, viable islets that exhibited strong intracytoplasmic immunopositivity for insulin, with a small proportion of islets exhibiting positive glucagon staining. Islets in the capillary alginate hydrogel preparation that were not associated with the hydrogel appeared fragmented, although they maintained positive insulin staining. A small number of cells stained positive for CC3. Immunohistochemistry (IHC) for proinsulin was considered negative with moderate, nonspecific, background staining of the hydrogel matrix material (Fig. 2D). Islets in the chitosan-based hydrogel preparation were mildly fragmented at d21 and were not associated with the hydrogel material. A small number of islet cells exhibited immunopositivity for CC3 and weak to moderate immunopositivity for proinsulin.

Islets in all devices had detectable C-peptide measured in outflow for the duration of the experiment. Islets that were transplanted with the capillary alginate hydrogel support had higher and more persistent levels of C-peptide measured in outflow as compared to the chitosan hydrogel and naked islet loaded devices (Fig. 2E). Capillary alginate hydrogel supported that islets had relatively stable C-peptide production for the first 30 days (3771 ± 671.1 ng/ml), following which levels decreased but remained detectable in outflow up to day 90 (102.6 ± 36.41 ng/ml). Chitosan-based hydrogel supported that islets not only had overall lower C-peptide production but also maintained detectable C-peptide levels in outflow up to day 90 (4.9 ± 1.4 ng/ml). Islets that did not have hydrogel support had similar levels of C-peptide in outflow as the capillary alginate hydrogel devices for the first 14 days, followed by a sharp decline (Fig. 2E).

Both islet-hydrogel constructs were glucose responsive, as indicated by glucose-stimulated insulin secretion (GSIS), with higher levels of C-peptide measured in the capillary alginate hydrogel constructs than the chitosan-based constructs (Fig. 2F). Gasses, pH, and electrolyte levels in the device media remained stable throughout the course of the study (table S1).

### Biocompatibility and safety of the CS in small and large animal studies

**Fig. 3.**
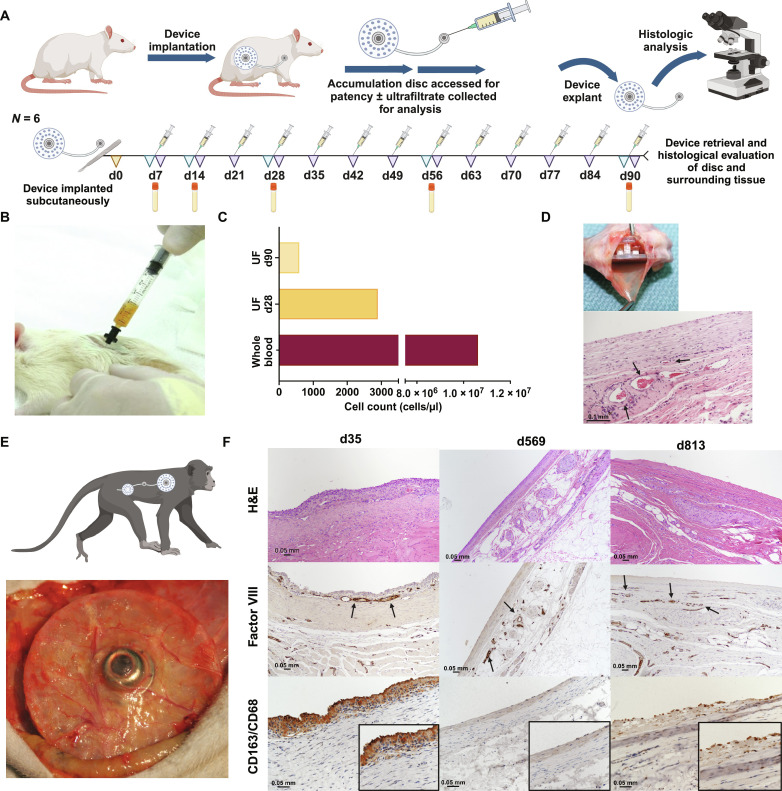
CS device biocompatibility studies in rats and NHPs. (**A**) Rats (*n* = 6) were implanted with the CS AD to assess device safety and biocompatibility, as well as assess UF composition. (**B**) Serial collection of UF was performed weekly using a standard 22-gauge Huber needle. (**C**) UF is depleted of cells as compared to circulating peripheral blood. (**D**) Fluid AD explanted from rats at 90 days. Representative histology of the fluid AD-tissue interface shows a mature, well-organized, moderately dense fibrous connective tissue with evidence of vascularization (black arrows). Scale bar, 0.1 mm. (**E**) Gross image of fluid AD implanted in NHP. (**F**) Representative histology of the AD-tissue interface in NHPs stained by hematoxylin and eosin, factor VIII (vascular tissue, black arrows), and CD163/CD68 (M2 macrophages, featured insets) at 35, 569, and 813 days following device implantation. Scale bars, 0.05 mm.

A 90-day biocompatibility and safety study was performed in healthy Lewis rats (*n* = 6) implanted with the AD attached to an access port for serial sampling of UF (Fig. 3, A and B). The device was well-tolerated with no adverse events associated with the device or protocol, skin overlying the device was normal, and weight gain was appropriate for age (fig. S2). Histologic evaluation of the device-tissue interface surrounding the AD was highly similar among the six rats with minimal tissue reaction to the device. The capsules were composed of mature, moderately dense fibrous connective tissue with evidence of vascularization with minimal inflammatory cell infiltrate at day 90 (Fig. 3, C and D).

Nine devices were tested in a total of seven NHPs (Fig. 1C and table S3) in a combined exploratory safety and efficacy assessment. One device was not loaded with islets due to a localized infection, and two devices met early endpoint criteria due to graft failure from contaminated islet product (table S3). Biocompatibility was evaluated in NHPs using the same criteria as previous work in rats, with similar findings at the device-tissue interface, demonstrating that the material response is highly reproducible. Photomicrographs show the orderly progression of interface formation surrounding the AD over time (Fig. 3F). The interface is composed of vascularized, well-organized, mature fibrous connective tissue (Fig. 3E). At early assessment, 35 days post-implant, the interface is composed of one to two cell layers of epithelioid macrophages overlying loose, lamellar layers of fibrovascular tissue. The chronic response, assessed at >1 year post-implant, is similar in appearance with the interface composed of dense, lamellar layers of collagen with the absence or minimal numbers of macrophages. Multiple vessels and nerves are appreciated subjacent to the well-organized fibrous capsule. Factor VIII IHC identifies endothelium lining vessels, and CD163/CD68 double IHC identifies diminishing numbers of macrophages, primarily polarized toward the M2 phenotype, lining or associated with the interface. Overall, the inflammatory cell presence is minimal and consists of few macrophages and lymphocytes mostly located perivascularly (Fig. 3F).

### UF characteristics in small and large animal studies

**Fig. 4.**
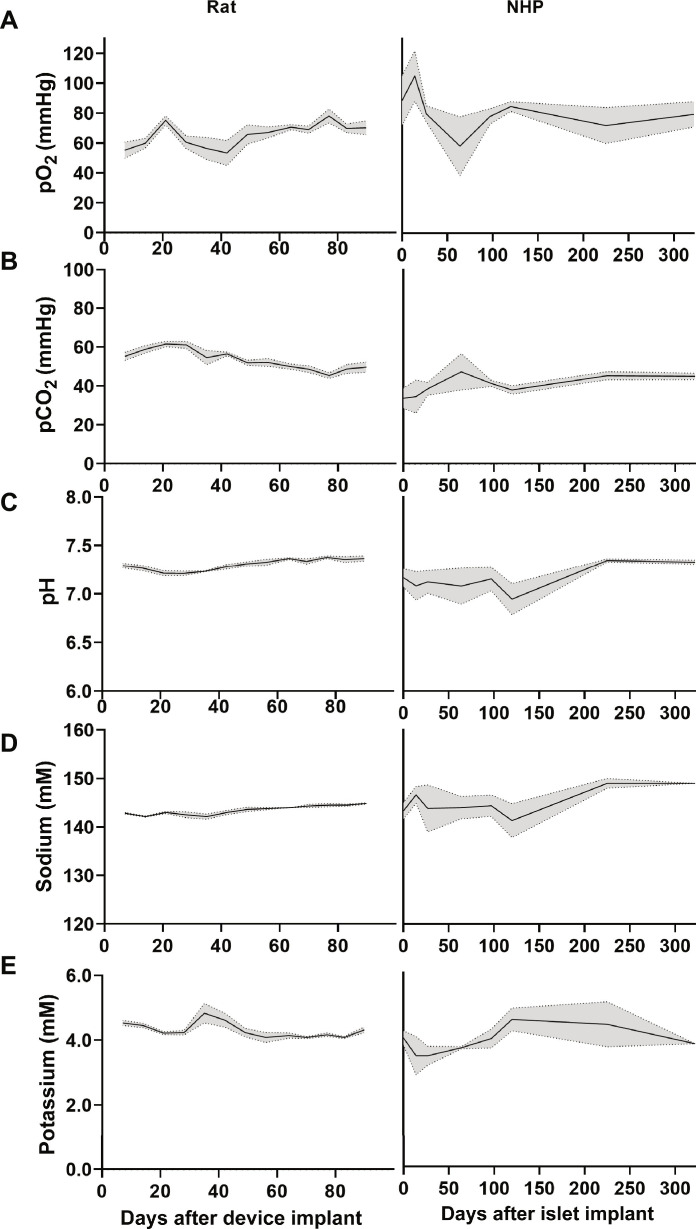
Mass transfer in CS device UF in rats and NHPs. UF in the CS AD was collected up to d90 in rats, without transplanted cells, and up to d300 in NHPs, with transplanted islets. UF trends in (**A**) pO_2_, (**B**) pCO_2_, (**C**) pH, (**D**) sodium, and (**E**) potassium. Data presented as mean ± SEM.

In rats, UF was collected from the AD implanted in the rat cohort (*n* = 6) to assess volume, composition, and stability over time. UF successfully accumulated in all devices by day 7 following implant through day 90. UF characteristics remained stable throughout the course of the 90-day study and closely resembled serum (table S2), with some notable differences, including slightly lower protein levels and low cellularity consistent with transudate. Oxygen levels in the UF remained stable (~60 mmHg) and avoided hypoxic conditions within the device (Fig. 4A).

In NHPs (*n* = 5), UF was sampled to evaluate gas and electrolytes following transplant. Oxygen levels within the UF remained stable and avoided hypoxia (Fig. 4A). UF characteristics closely resembled those of peripheral blood (Table 1), along with a neutral pH and normal electrolyte composition, but with a slightly lower protein profile (Table 1).

**Table 1.** Characterization of CS-generated UF in NHPs. Comparison of UF generated by the AD before islet graft loading with paired peripheral blood samples taken in parallel (*n* = 4). Data are presented as mean ± SEM.

	UF	Peripheral blood	Human reference range
Urea nitrogen (BUN) (mg/dl)	14.3 ± 2.6	13.0 ± 1.9	8–20
Creatinine (mg/dl)	0.8 ± 0.1	1.0 ± 0.1	0.5–1.2
Calcium (mg/dl)	7.4 ± 0.5*	9.5 ± 0.3	9–10.5
Phosphorous (mg/dl)	3.6 ± 0.2	3.6 ± 0.4	3–4.5
Magnesium (mg/dl)	1.7 ± 0.2	1.9 ± 0.2	1.5–2.4
Total protein (g/dl)	3.7 ± 0.5*	6.8 ± 0.4	6–7.8
Albumin (g/dl)	2.2 ± 0.3*	4.1 ± 0.2	3.5–5.4
Globulins (g/dl)	1.5 ± 0.2*	2.7 ± 0.2	2.5–3.5
Sodium (mM)	146.3 ± 0.8*	144.3 ± 1.0	136–145
Chloride (mM)	111.1 ± 2.1	109.3 ± 1.0	98–106
Potassium (mM)	3.6 ± 0.2	3.6 ± 0.2	3.5–5
Bicarbonate (mM)	16.4 ± 1.4*	24.5 ± 0.4	23–29
Osmolarity (calc.)	289.5 ± 1.5	290.8 ± 2.6	275–295
Total bilirubin (mg/dl)	2.3 ± 0.7	0.2 ± 0.0	0.3–1.2
Alkaline phosphatase (U/liter)	39.75 ± 6.3*	126.5 ± 15.7	36–150
Aminotransferase, alanine (ALT) (U/ liter)	14.0 ± 2.3	34.6 ± 7.3	<35
Aminotransferase, aspartate (AST) (U/ liter)	44.0 ± 11.3	19.3 ± 1.9	<35
Creatine kinase (CK) (U/liter)	103.5 ± 68.9	301.0 ± 159.3	30–170
Glucose (mg/dl)	71.0 ± 13.0	158.8 ± 58.8	60–99^†^
Cholesterol (mg/dl)	74.5 ± 38.9*	128.0 ± 8.7	<200
Amylase (U/liter)	78.0 ± 19.3*	153.5 ± 24.4	<110
pH	7.2 ± 0.1		7.35–7.45
pCO_2_ (mmHg)	33.7 ± 5.2		35–45
pO_2_ (mmHg)	88.8 ± 16.6		80–100

*Paired *t* test *P* < 0.05.

^†^Nondiabetic range.

Trends in gases and electrolyte levels in UF remained largely stable throughout the course of the study in both NHPs and rats (Fig. 4). Comparison of UF characteristics between CS and CS-h devices implanted in NHPs remained similar despite differences in active versus passive convection mechanisms (fig. S3). Devices implanted in NHPs were maintained past the point of graft failure to determine the persistence of UF generation and characteristics, and UF was maintained >1 year in multiple recipients.

### Immune protection of transplanted cells within the CS

**Fig. 5.**
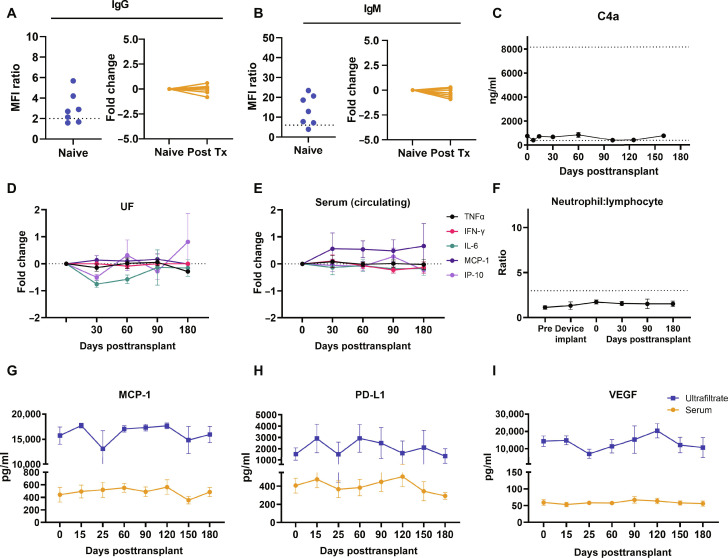
Immune protection of transplanted cells within the CS device in NHPs. (**A** and **B**) Assessment of donor-specific antibodies did not indicate an immune response to the islet graft within the CS device, despite high reactivity before graft loading. Data presented as mean fluorescence intensity (MFI) in the naive state and posttranplant (Post Tx). (**C**) Complement activation measured in UF remained within normal limits [normal value range indicated by dotted line (*108*); *n* = 7]. Proinflammatory cytokine levels remained stable both (**D**) within the CS device and (**E**) in circulating serum up to 180 days after islet transplant (*n* = 5). (**F**) Neutrophil-lymphocyte ratio remained within normal limits (indicated by dotted line) following device implantation and islet cell loading. Levels of (**G**) MCP-1, (**H**) PD-L1, and (**I**) VEGF were measured in both peripheral serum and device UF, with levels within the device remaining higher than that in the periphery. Data in (C) to (I) presented as mean ± SEM. TNFα, tumor necrosis factor–α; IFN-γ, interferon-γ; IL-6, interleukin-6; interferon gamma-induced protein 10 (IP-10).

All NHP recipients demonstrated high reactivity analyzed by donor-specific antibody flow crossmatch that would normally preclude eligibility for conventional intraportal islet xenotransplant (Fig. 5, A and B), modeling a patient population that has low eligibility for transplant and high risk of rejection. Serial biopsies from devices implanted in NHPs were scored by a pathologist for immune cell infiltration (table S5).

In 58 of 58 biopsies, there was no evidence of inflammatory cells present in the graft. Considering the potential for complement-dependent toxicity to islets, IHC staining for immunoglobulin G (IgG) and IgM was performed on islet biopsies from the CS devices 30 days following transplantation, which showed no immunoreactivity for IgG or IgM within the biopsy (fig. S4). Similarly, the complement subunit C4a, selected for its stability over time, was measured in UF demonstrating no activation of complement (Fig. 5C). The assessment of donor-specific antibodies posttransplant did not indicate an immune response to the porcine islets transplanted in the device, suggesting successful immune isolation of the therapeutic cells (Fig. 5, A and B). Together, this suggests a stable nonreactive immune environment within the device and no evidence of increased systemic inflammation in response to xenogenic cells housed within the device.

In both rats and NHPs, the presence of immune cells observed in UF was minimal. In the rat study, UF was evaluated over time for cellular composition (Fig. 3C) and found to be largely deplete of immune cells. The presence of inflammation was further profiled in a multiplex cytokine panel to evaluate expression in both UF and circulating serum. Comparison of the fold change of proinflammatory cytokines in UF and the periphery showed no significant changes over 180 days (Fig. 5, D and E). The neutrophil-to-lymphocyte ratio remained within normal limits throughout the duration of the islet graft (Fig. 5F). Measured cytokines showed elevated levels of monocyte chemoattractant protein-1 (MCP-1) (Fig. 5G), programmed death-ligand 1 (PD-L1) (Fig. 5H), and vascular endothelial growth factor (VEGF) (Fig. 5I), within the device as compared to the periphery, indicative of a wound healing environment.

### Effectiveness of the CS or CS-h bioengineered artificial interstitium for xenogeneic porcine islet transplant in non-immunosuppressed NHPs

In the first implant of the CS device in a nondiabetic NHP, follow-up was intentionally limited to 30 days, even with persistent C-peptide measured in outflow, to enable assessment of the device-tissue interface at an early time point before attempting to extend follow-up. In the subsequent six NHPs, the survival and function of transplanted islets were compared between the CS and CS-h in two additional nondiabetic (total *n* = 3) and diabetic (*n* = 4) at low- to high-density preparations with hydrogel and without (Fig. 1C). Porcine C-peptide was routinely measured in outflow UF as an in-phase marker of graft survival, defined as the last measured day > 0.5 ng/ml, as measured by enzyme-linked immunosorbent assay (ELISA). Kaplan-Meier survival analysis showed a higher rate of graft survival in the CS bearing hydrogel constructs than the CS-h bearing only naked islets at both 3 and 6 months (100% versus 75% and 100% versus 37.5%, respectively) demonstrating that both devices were capable of supporting long-term survival of transplanted cells (Fig. 1D). Since only naked islets were loaded into CS-h devices related to the possibility that hydrogel-islet constructs might increase resistance in a passive flow situation, the survival analysis by loading condition is identical to device condition. There was no apparent relationship between islet dose/density and duration of survival; however, hydrogel support appeared to increase the stability and level of insulin secretion.

**Fig. 6.**
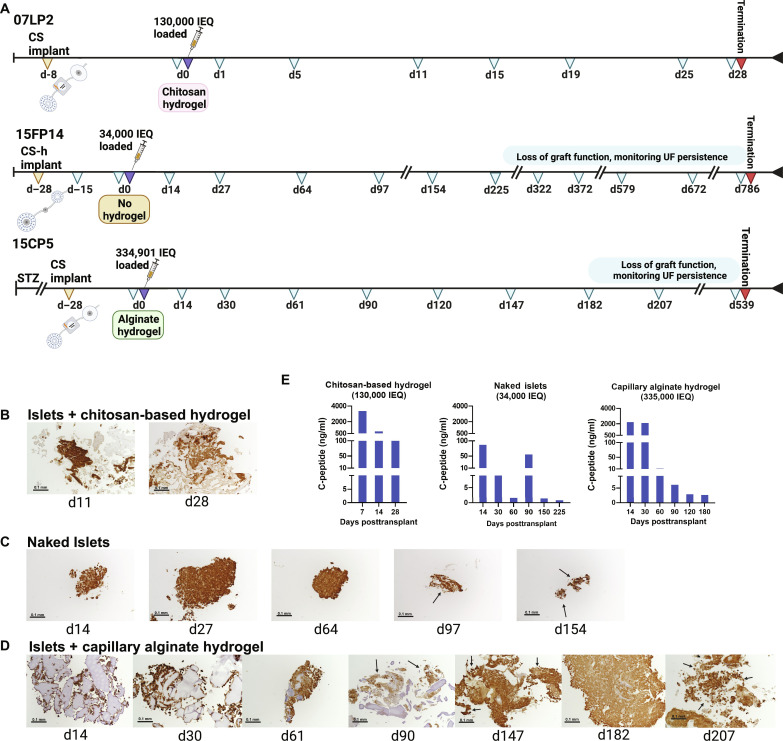
Long-term health and function of transplanted islets in the CS device in NHPs. (**A**) Timeline of device implantation, cell transplantation, and sampling. Representative biopsies of islets transplanted with (**B**) chitosan-based hydrogel support, (**C**) no hydrogel support, and (**D**) capillary alginate hydrogel support. All biopsies are stained for insulin. Naked islets and islets with capillary alginate hydrogel support demonstrate fragmentation starting at d97 and d90, respectively, but continue to demonstrate positive insulin staining. Arrows point to areas of islet fragmentation. Scale bars, 0.1 mm. (**E**) Graft function measured by C-peptide expression in UF up to 180 days following islet transplantation.

Serial islet biopsies under each condition were evaluated for morphology, viability, infiltration, and insulin (Fig. 6A). In the short-term study, a chitosan-based hydrogel was used as an injectable scaffold for transplant of 130,000 IEQ to the CS device, where C-peptide levels > 100 ng/ml were detected in UF for 30 days and serial biopsies confirmed the presence of insulin-positive cells (Fig. 6, B and E).

In CS-h devices transplanted with naked islets, the longest duration of survival as measured by porcine C-peptide > 0.5 ng in UF was 225 days with biopsies demonstrating insulin-positive cells up to day 150 (Fig. 6, C and E). In the CS device transplanted with a capillary alginate hydrogel-islet construct, porcine C-peptide was >0.5 ng in UF up to 182d with biopsies demonstrating insulin-positive cells up to d200 (Fig. 6, D and E). Islets showed an increase in fragmentation over time, with β cells adhering to hydrogels remaining insulin positive (table S6), consistent with the slow decline in the C-peptide level.

**Fig. 7.**
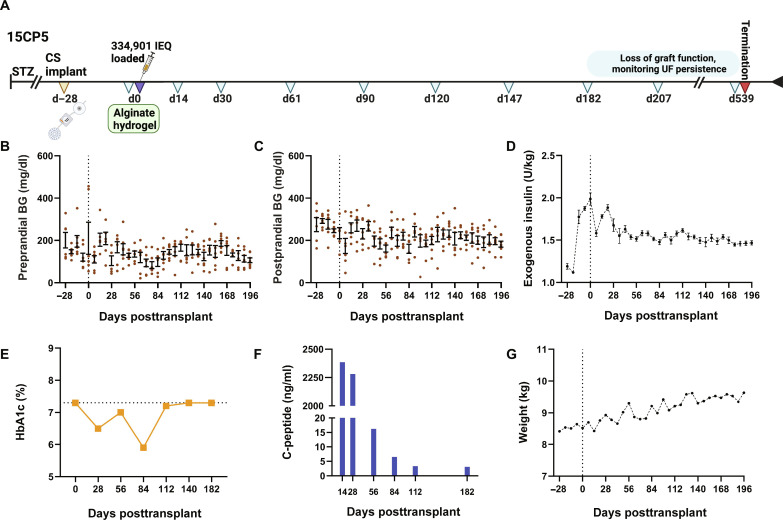
Long-term metabolic effects of transplanted islets in the CS device in diabetic NHP 15CP5. (**A**) Study timeline of 15CP5. Daily measures of (**B**) preprandial and (**C**) postprandial blood glucose (milligrams per deciliter) by day posttransplant of 334,901 IEQ capillary alginate supported porcine islet cells into a CS device (data presented as mean ± SEM). (**D**) Average daily exogenous insulin requirement (units per kilogram) by day posttransplant. (**E**) HbA1c (%) and (**F**) porcine C-peptide (nanograms per milliliter) detected in device outflow by day posttransplant. (**G**) Weight by day posttransplant.

Metabolic effects of grafts within the CS devices were assessed in diabetic animals through long-term measurement of preprandial and postprandial blood glucose and hemoglobin A1c (HbA1c) values and C-peptide production by the grafts, all in relation to exogenous insulin administered to the animals. Recipients transplanted with islets in the CS and CS-h realized the most favorable glycemic control during periods where consistent levels of porcine C-peptide secretion were measured, with a resulting transient reduction in HbA1c (Fig. 7 and figs. S5 and S6), even when a minimal mass, as low as 34,000 IEQ, was transplanted.

### Effectiveness of the CS or CS-h bioengineered artificial interstitium for xenogeneic porcine islet transplant in non-immunosuppressed NHPs previously sensitized to pig

**Fig. 8.**
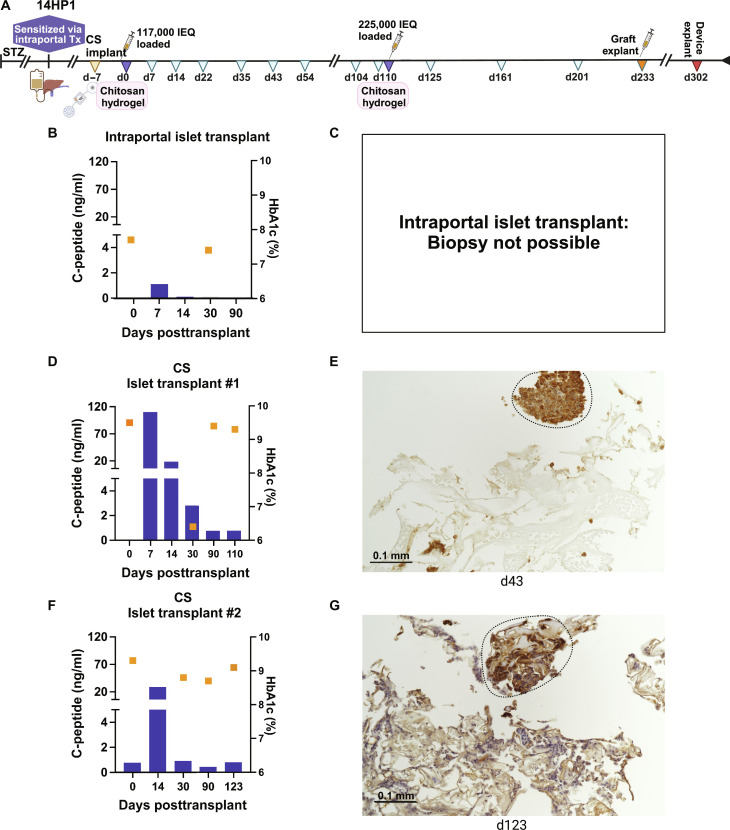
CS device protects graft against a sensitized recipient (14HP1). (**A**) One NHP that was implanted with a CS device had previously been transplanted with intraportal porcine islets. Graft function as measured by C-peptide (blue bars) in the device and HbA1c% (gold squares), along with representative islet biopsies stained for insulin are presented for (**B**) and (**C**) the initial intraportal transplant (157,500 IEQ), (**D**) and (**E**) CS transplant #1 (117,000 IEQ), and (**F**) and (**G**) CS transplant #2 (225,000 IEQ). Dashed lines highlight representative islets. Time points with no detectable C-peptide are indicated by the absence of blue bars. Scale bars, 0.1 mm.

Immunoisolation of the graft within the CS device was tested in two animals intentionally sensitized to pigs using conventional intraportal islet transplant under the cover of high-dose rapamycin monotherapy, as exemplified in Fig. 8A. Following intraportal islet transplant, porcine C-peptide was >0.5 ng/ml for 7 days (Fig. 8B). The CS device was implanted and successfully loaded with two subsequent porcine islet products without use of any immunosuppression, surviving for >110 and >123 days, respectively, as indicated by C-peptide levels > 0.5 ng/ml in UF and confirmed by insulin-positive cells during biopsy (Fig. 8, E and G). Similar to nonsensitized animals, HbA1c% reduction was most appreciable following periods of detectable porcine C-peptide (Fig. 8, D and F).

## DISCUSSION

The CS and CS-h devices successfully mimic interstitium creating a unique microenvironment capable of supporting and protecting transplanted cells. UF is naturally deplete of the typical immune cell repertoire so that transplanted cells are not exposed to normal immune cell presentation. The convective action of the device circulates UF, which serves as a transport medium for gas, nutrients, and waste products as well as signaling proteins produced locally or circulating systemically and transports expressed therapeutic proteins to the recipient.

Serial percutaneous needle biopsies as a feature of the device allowed direct investigation of the UF to characterize the microenvironment as well as longitudinal assessment of the graft. Long-term continuous stable accumulation of UF was achieved in both rodents and NHPs with normal oxygen, pH, and chemistry and a favorable cytokine profile for graft survival and function. As the tissue-device interface surrounding the AD matures and integrates with the surrounding capillary and lymphatic channels, oxygen, CO_2_, and pH levels stabilize and remain within a relatively narrow range even following cell transplantation. The pO_2_ levels are generally >60 mmHg, avoiding hypoxia that cells would typically be exposed to in the subcutaneous space. Using adult pig islets, we have demonstrated that an artificial interstitial environment can support xenograft cell survival for more than 180 days in non-immunosuppressed NHPs. There has been some success with macroencapsulation of xenogenic islets; however, most approaches require invasive techniques such as vascular anastomoses or intraabdominal placement, complicating surgical graft retrieval and retransplant (*3*). Diabetes has been corrected in NHPs using high dose (30,000 IEQ/kg) of porcine islets encapsulated in an alginate pouch placed subcutaneously, without immunosuppression (*59*). In this study, the islet cell mass required and their decline in function over time highlights the need for a scalable approach toward translation as well as the possibility to reload islets without surgical removal and replacement of the device.

Adult pig islets were chosen with the perspective that xenotransplantation offers a potentially unlimited source of cells for islet grafts with a well-known insulin kinetic profile. Stem cell–derived therapies, including genetically modified lines, have equal potential for success in a niche environment and promise as an unlimited cell source with the potential advantage of less fragility as compared with isolated islet cell products.

### Biocompatibility and safety

The CS and CS-h are primarily composed of silicone that is chemically inert, durable, and widely used as the material of choice for medical devices with a long track record of biocompatibility and safety. In rodents (Fig. 3D) and NHPs (Fig. 3E), the devices exhibited a minimal foreign body reaction. Histologic evaluation of the device-tissue interface demonstrated a physiologically appropriate progression toward wound healing and fibrovascular capsule formation; specifically, mature, moderately dense fibrous connective tissue with the minimal inflammatory cell presence consisting of few macrophages, primarily polarized toward the M2, or tissue repair, phenotype, as well as minimal lymphocytes near blood vessels (Fig. 3F) in NHPs. There was an impressive degree of vascularization around the AD (Fig. 3E). Because the entire device is implanted in the subcutaneous space and relies on extravasated fluid as opposed to a direct vascular connection, there is no need to expose the recipient to systemic anticoagulation, improving safety. This similarly allows for easy explantation of the device should it be necessary.

Nine devices were tested in seven NHPs; however, one device was explanted due to localized infection without having islets loaded, whereas two devices met early endpoint criteria for graft failure subsequently found to be the result of contaminated islet product. In human islet transplantation, microbial contamination of islet preservation media is a known problem associated with poorer islet yield and lower C-peptide/insulin independence rates (*60*) with some contamination rates reported as high as 64.4% of human islet donors (*61*). This highlights the importance of ensuring sterility during islet handling. For the device with a localized infection, as is well known with implanted devices and surgery in general, surgical site infections do happen; however, because of the subcutaneous location, removal was relatively simple with minimal burden to the animal compared to devices implanted intraabdominally or with a vascular connection requiring a much more involved removal.

### UF as a perfusate for transplanted cells

Islet cells are particularly sensitive to hypoxia, requiring high levels of oxygen to survive (*62*, *63*). Recognizing this, different devices and techniques have attempted to address the need for higher local oxygen levels for islet survival for more than 30 years. In the 1990s, Monaco and Sullivan used a perfusion-based, vascularized bioartificial pancreas in dog models for the treatment of diabetes. Although demonstrating efficacy, these devices required invasive surgery with vascular anastomoses and ultimately were not successful in vivo due to high rates of synthetic vascular conduit thrombosis (*64*, *65*). Recently, a BioVascular Pancreas was created using an acellular vascular graft as a scaffold for islet cells allowing close contact with fully oxygenated arterial blood—an approach that requires invasive vascular surgery and immunosuppression (*66*). The βair Bio-Artificial Pancreas increases local oxygen availability by directly supplying oxygen via daily injection through a subcutaneous port to the housed islets (*67*, *68*). Even in conventional intraportal transplant, portal blood pO_2_ is typically ~40 mmHg exposing islets to hypoxia at transplant (*69*, *70*). This low oxygen tension, along with IBMIR seen in portal transplant, results in an estimated loss of up to 70% of the graft before capillary revascularization (*71*, *72*). In contrast, the AD was capable of generating UF as an autologous oxygen source in both rodents and NHPs with measured pO_2_ levels of >60 mmHg and stable pCO_2_. The pH was within a narrow range in in vitro studies, implanted rodents, and CS-h implanted NHPs, with variations in pH specific to CS-implanted NHPs. This suggests that pH may have been affected by the volume, composition, and degradation of hydrogel since this was exclusively used in CS-implanted NHPs. It has been shown that islets cultured at pH levels between 6.5 and 8.0 maintain the ability to release insulin short term, with the best preservation of β cell function found at pH 7.2 long term in culture (*73*). The extensive vasculature that develops around the AD and high gas permeability of the silicon-based AD and cell house allow for this gas exchange with the surrounding tissue.

### Immune protection

Testing immunoisolation was a primary motivation for this work. The CS and CS-h were designed with the intent to protect and isolate transplanted cells from the recipient immune system making immunosuppressive drugs unnecessary which shifts the risk-benefit profile for patients undergoing transplant. The relatively large pore size of the filter in the cell house was to minimize resistance to the flow of UF and simultaneously assure transplanted cells or cell-hydrogel constructs are contained within the chamber. A small number of immune cells can be found in UF (Fig. 3C); however, there was no evidence of islet cell infiltration on histologic analysis of serial islet biopsies.

IgG/IgM staining of islets recovered from biopsies did not suggest the presence of antibody deposition on graft cells despite all recipients demonstrating highly reactive donor-specific antibodies. It may be that the cell house limits xenograft antigen in the outflow from releasing into the interstitium and stimulating antibody production by immune cells. The presence of M2 macrophages on the device-tissue interface favors an immunoregulatory environment and supraphysiologic levels of VEGF, an immunosuppressive factor (*74*), in UF (Fig. 5I) can directly modulate immune cells to prevent antibody binding to islets (fig. S4).This was consistent with findings in peripheral blood when evaluated for donor-specific antibodies, which had no appreciable increase in the posttransplant period. While serial biopsies of islets within the devices indicated progressive islet fragmentation over time, there was no evidence of complement activation, suggesting a mechanical driver for islet disaggregation rather than complement-mediated cytotoxicity.

Without immunosuppression, xenogeneic islets are typically destroyed within 7 days in the pig-to-NHP model. The device not only prevented graft immune cell infiltration but also averted systemic changes related to xenograft transplant, especially inflammation. Cytokine levels remained stable both within the device and in circulating serum and with no elevation of neutrophil-lymphocyte ratio. The increased levels of MCP-1, VEGF, and PD-L1 in UF as compared to serum likely reflect chronic tissue remodeling that is provoked by the design of the AD where macrophages can be seen at the periphery of the device-tissue interface. MCP-1 and PD-L1 are produced by macrophages, and absent other changes in proinflammatory cytokines are likely polarized toward the suppression of inflammation to facilitate tissue repair during healing. Furthermore, VEGF has been shown to be important for maintenance of adult islet cells, and an increase in VEGF expression has been shown to increase graft revascularization after islet transplantation; therefore, the increased levels of VEGF in UF is likely of benefit to the transplanted cells (*75*, *76*). Cells survived for more than 30 days without immunosuppression even in sensitized animals.

### Graft survival and function

In NHPs, we demonstrated that islets could be transplanted into the CS and CS-h with or without hydrogel from low to high density and observed long-term xenograft survival and functionality measured both by porcine C-peptide and positive insulin at biopsy. Insulin secretion was generally greater at higher islet doses as was expected; however, all of the transplants suffered a decline in insulin production, most prominently after the first 30 days regardless of dose or condition (± hydrogel). In diabetic NHPs, the presence of positive porcine C-peptide conferred at least modest metabolic benefit with some animals realizing improvements in HbA1c reduced by more than 1% that had not been achieved with exogenous insulin alone (Fig. 7). While NHP recipients did not achieve insulin independence related to insufficient insulin secretion, especially as islet cells disassociated to individual β cells, we were able to demonstrate the practicality of generating UF for the creation of an artificial interstitium as a site for cell transplantation.

Islet cell morphology was affected by hydrogel construct and transplant duration, naked islets demonstrated fragmentation (Fig. 6, C and E) earlier than islets delivered with hydrogels (Fig. 6, D and E), and even under carefully controlled in vitro conditions, perifusion resulted in eventual islet disaggregation. It should be noted that assessment of needle aspirates for islet morphology may favor sampling of fragmented islets and introduce some fragmentation via the sampling process, as the biopsy is aspirated by gently agitating the graft to obtain a sample. In addition, adult porcine islets are fragile as a result of the isolation process and have some degree of fragmentation even in static cultures (*77*). Native pancreatic islets interact with neighboring cells via paracrine and autocrine interactions (*78*), and loss of extracellular matrix (ECM) in the isolation of islet cells leads to anoikis, a form of programmed cell death (*79*, *80*). Given this, the fluid environment mostly devoid of ECM components within the device may contribute to islet disaggregation. While perifusion has been shown to improve the microenvironment by mimicking in vivo physiology, this is dependent on more than just flow conditions (*81*). There was improvement in graft survival with the use of the capillary alginate hydrogel as a scaffold, selected for its unique properties of customizable open channels throughout, and even appeared to support some degree of reaggregation along channels (Fig. 6D). Further work is needed to develop the optimal supportive matrix that can advantage the device characteristics, that is, gels that are injectable and retrievable via a needle. It has also been reported that during the isolation of porcine islets, the cleavage process results in islets of heterogeneous sizes and morphology making porcine islets particularly fragile compared to human islets (*82*). Convection-based devices may introduce different challenges for preparing the graft than conventional diffusion-based macrodevices. This approach has the major advantage of overcoming the typical diffusion restrictions afflicting macroencapsulation devices; however, flow-based devices exert sheer damage to cells. As a first in NHP study of such an approach, the major aim to extend graft survival beyond what has been observed without immunosuppression was the focus, and as a result, this study did not optimize flow conditions, so it remains unclear what the optimal flow rate is to prevent sheering. While porcine islets may be particularly fragile, other cell types used in cell-based therapies are likely more robust and may not experience the same degree of loss of integrity under similar conditions.

### Patient compliance

Patient compliance is a critical factor to the success of any therapeutic device. Patients with T1DM are approximately two to three times more likely to die (*83*) and have a life expectancy 10 to 13 years less than the general population (*84*, *85*). A history of insulin treatment noncompliance in patients with T1DM is associated with an increased all-cause mortality as well as an increased likelihood of having increased BMI and HbA1c at baseline (*83*). The CS and CS-h may have considerable benefit in this regard over traditional insulin pumps. Insulin pumps come in a variety of designs but most often require refilling of insulin or are disposable requiring replacement or refilling every 1 to 3 days, and tethered pumps often require daily charging of the battery (*86*, *87*). In contrast, the CS device requires approximately weekly charging via induction, while the CS-h exploits hydrostatic pressure and therefore does not require any charging. Vulnerabilities in software and incorrect insulin algorithms present potential safety issues (*88*). This is not an issue in devices bearing islet cells that are capable of sensing and releasing insulin as needed, not relying on software or sensors to evaluate blood glucose levels.

### Limitations of the study

This is the first evaluation of this concept, and the exploratory nature of the work NHP study includes a small number of subjects. While this allowed for the flexibility to experiment with different approaches to determine device characteristics that proved innovative and relevant, all aspects that may be relevant were not systematically evaluated (e.g., dose dependency and flow characteristics) which may be studied more in depth in future studies now that basic hurdles related to immunosuppression and short-term durability have been established.

### Application beyond islet transplantation

The CS and CS-h have demonstrated their ability to support and immunoprotect xenogenic islet cells; therefore, it is logical to assume that this technology can be applied to other cell-based therapies. Many cell-based therapies rely on direct delivery of cells to the target site to enact function, for example, the putamen in Parkinson’s disease (*7*, *89*) for dopamine production or the cardiac myocardium in cardiac ischemia (*90*, *91*) for cardiac repair. Conversely, in endocrine disorders, the disease state is due to a lack of systemically circulating hormone or product; therefore, function is not dependent on cell engraftment location as long as the hormone can reach circulation. With this in mind, endocrine cell-based therapies to treat adrenal insufficiency, hypothyroidism, or hypoparathyroidism could advantage the CS and CS-h bioengineered artificial interstitium for transplant.

For adrenal insufficiency, a growing body of research is emerging in regard to the future treatment and management of the disorder. Similar to islet cell transplantation, adrenal cell transplant faces the challenges of chronic immunosuppression and having a supportive environment to facilitate ongoing function. This approach could facilitate immunoisolation of adrenal cells without the need for chronic immunosuppression and interaction with physiologic drivers such as adrenocorticotropic hormone, angiotensin II, and potassium. Likewise, endocrine cell therapies for hypothyroidism and hypoparathyroidism could be a target. Recent studies have shown that stem cells can be induced to become functional thyroid follicular cells (*92*, *93*) but do require organization into a 3D follicle-like structure for enhanced functionality. Injectable hydrogel matrices could provide such a scaffold for these cells in the CS or CS-h devices. Parathyroid cell allotransplantation and stem cell therapy (*94*) for hypoparathyroidism face similar challenges as previously discussed endocrine cell therapies.

Aside from these endocrine cell-based therapies, the CS and CS-h would be advantageous with other stem cell–based therapies due to their retrievability and reservoir access. The tumorigenic potential of transformed or gene-modified cells continues to be of particular concern as these therapies progress through the translation phase. The generation of iatrogenic tumors or teratomas derived from human stem cells, including human embryonic stem cells (hESCs) and induced pluripotent stem cells (iPSCs), has been discussed at length in the literature (*5*, *9*, *13*, *95*), highlighting the need for easy access, monitoring, and removal of these cells. The CS and CS-h provide simple percutaneous access to the reservoir allowing for easy extraction or monitoring of these cells. Furthermore, if concern for teratoma or tumor formation necessitated, then the entire device could be retrieved from the subcutaneous space with relative ease.

We report a method of bioengineering the subcutaneous space into an artificial interstitium using the silicone-based CS and CS-h devices for xenogeneic islet cell transplantation in non-immunosuppressed NHPs. This approach was durable, safe, and efficacious with insulin production in a subset of recipients up to 6 months and more than 3 months in a sensitized transplant recipient. Furthermore, positive impacts on glucose control were seen in diabetic NHPs concordant with levels of insulin production. Different from encapsulation strategies or devices that rely on diffusion for transport, this approach is dynamic using autologous UF for gas exchange, nutritional support, removal of waste, and distribution of signaling molecules. The subcutaneous location allows for ease of implant and device removal, while the percutaneous accessibility permits reloading, biopsy, and removal of cells. This demonstrates long-term survival of xenogenic islet cells in an NHP model without immunosuppression, using percutaneous noninvasive access for graft loading, reloading, or retrieval to our knowledge. While this work was aimed toward cell transplantation for T1DM, given these attributes, this device platform has potential for use in a wide variety of regenerative medicine applications using cell-based therapies.

## MATERIALS AND METHODS

### Animal subjects

All animal procedures were approved by the University of Minnesota Institutional Animal Care and Use Committee, conducted in compliance with the Animal Welfare Act, adhered to principles stated in the National Institutes of Health Guide for Care and Use of Laboratory Animals (*96*) and were performed and reported in compliance with the Animal Research: Reporting of In Vivo Experiments (ARRIVE) guidelines. All animals were purpose-bred and purchased from institutionally approved commercial vendors. Animals used in this study were randomly assigned to study group/experimental condition based on availability and the current phase of the study; because of the study’s purpose and exploratory nature, no animals were assigned to a conventionally defined control group. Because of clinical care requirements, experimenters could not be blinded to an animal’s experimental condition for certain aspects of the experiment including metabolic characterization. Blinding occurred during data analysis when feasible.

### CS and CS-h devices

The CS device is a three-component device designed to be implanted subcutaneously to provide isolation, nutrition, and oxygen to transplanted cells while also allowing for individual component exchange or total device exchange. The three components consist of an accumulation chamber for the accumulation of interstitial fluid; an electromechanical pump to transfer the interstitial fluid from the accumulation chamber to the isolation chamber; and an isolation chamber (the “cell house”) to house the transplanted cells of interest (Fig. 1).

The accumulation chamber consists of three parts constructed from biocompatible silicone; top and bottom disks and an outlet catheter (fig. S1A). The top and bottom disks are molded from commercially available liquid silicone rubber (LSR). The top disk has a center “dome” designed to increase the volume of fluid that can be accumulated while also indicating whether the accumulation chamber is full or empty. The bottom disk consists of a plurality of posts spaced at such a distance to inhibit the ingrowth of tissue. In addition, the bottom disk has a drain via which the interstitial fluid can be pulled into the pump. The top and bottom disks are bonded together using a medical-grade room temperature vulcanizing (RTV) silicone. Last, a silicone outlet catheter is attached with medical-grade RTV to the drain area on the bottom disk for connection to the pumping mechanism (fig. S1A). For implant in nondiabetic rats, the AD was modified to 1.3-inch diameter × 0.5-inch height and implanted as a single component to assess basic biocompatibility, durability, and interstitial fluid composition as part of the efficacy and safety evaluation of this approach.

The electromechanical pump is composed (from bottom to top) of a rechargeable lithium-ion battery, a printed circuit board (PCB) outfitted with electronic components, ferrite sheeting, a micropump, and a wireless recharging coil. The battery, micropump, and recharging coil are connected to the PCB with the ferrite sheeting sandwiched between the wireless coil and the PCB. The components are further held together by a layer of Kapton tape. After testing and debugging, a set of tabs are removed from the PCB, and decals are applied to the top and bottom of the pump to aid in pump identification and flow direction. Next, tubing is attached to the inlet/outlet of the micropump, and the pump assembly is placed in a silicone mold, potted with a medical-grade epoxy, and allowed to cure for 24 hours. After removal from the epoxy mold, all sharp edges are ground, and the epoxy surface is thoroughly cleaned. The pumps are then dip-coated in a medical-grade, biocompatible silicone dispersion and allowed to cure for 24 hours. Last, the tubing used during the dip coating process is removed and replaced with a radiopaque, medical-grade silicone tubing. A fillet of medical-grade RTV is placed at the tubing to pump junction and allowed to cure for 24 hours (fig. S1B).

The isolation chamber consists of five parts; a top piece with an embedded access port, a bottom piece, a sintered titanium (Ti) filter, an inlet catheter, and an outlet catheter (fig. S1C). The modified access port is over-molded with a medical-grade LSR to create the top piece. Catheter inlet/outlet holes are punched into the top piece for eventual attachment of the inlet/outlet catheters. Similarly, the bottom piece is molded from medical-grade LSR. A sintered Ti filter is bonded into the bottom piece with medical-grade RTV and allowed to cure for 24 hours. The sintered Ti filter was designed with two main goals: (1) to limit resistance to fluid flow through the system and (ii) to prevent transplanted cells from escaping the isolation chamber. Then, the top piece is bonded to the bottom subassembly with medical-grade RTV. The two halves are tightly clamped and allowed to cure for 24 hours. Last, the inlet/outlet catheters are inserted into the inlet/outlet holes created earlier, and a fillet of medical-grade RTV is placed around the inlet/outlet catheters and allowed to cure for 24 hours.

Following the completion of the three main components and any subsequent verification testing, the three components can be connected for device testing (fig. S1D). The components are packaged and sterilized individually for eventual assembly at the study site. The pump is controlled via a software interface that allows for calibration, flow rate adjustments, charging status, report generation, and other various performance parameters. The >2-inch dimensions of the device (Fig. 1) precluded testing in a small animal model.

The CS-h device is a modification of the CS device, without the electromechanical pump component. Constructed using the same methods as the CS, the isolation chamber (cell house) is embedded within the AD. The CS-h exploits the hydrostatic and osmotic pressure gradient to drive convective flow of UF into the AD, through the cell house and out the outlet catheter. The >2-inch dimensions of the device (Fig. 1) precluded testing in a small animal model.

### Hydrogels

#### Capillary alginate gel (Capgel, C-G, Orlando, FL)

Capgel is a semisolid hydrogel that has numerous patent capillary microchannels running in parallel throughout (*97*). The gel self-assembles during the diffusion of Cu ions into alginate solutions and can be injected through a fine gauge needle in the form of a slurry to target sites.

#### Chitosan-based gel (Islet-Mate, BRTI Life Sciences, Two Harbors, MN)

Islet-Mate is a chemically defined material composed of chitosan and dextran sulfate. The formulation is initiated by exposing lyophilized chitosan to a solution of dextran sulfate containing a suspension of the cell population of interest, which are then distributed throughout the volume of the resulting hydrogel, which forms at room temperature.

### Swine

Adult swine served as islet donors for this study. Nineteen designated pathogen-free adult Mangalitsa porcine donors (18 sows, 1 gilt), aged 1.2 to 4.6 years old and weighing 183.2 to 262.7 kg, and one designated pathogen-free adult Berkshire sow donor, aged 3.5 years old and weighing 267.3 kg.

#### Islet isolation and quality control

Adult porcine islets were isolated and cultured as previously described (*98*, *99*) and evaluated for conventional quality control (purity, sterility, and viability assessed by oxygen consumption rate normalized for DNA) (*100*).

### In vitro device stress testing

Isolated adult porcine islets were cultured with or without a hydrogel bioscaffold and loaded at either low (<40,000 IEQ), mid (40,000 to 150,000 IEQ), or high (>150,000 IEQ) doses into the isolation chamber of the CS device. Once loaded into the device isolation chamber, islets were maintained for up to 90 days using culture media optimized for porcine islets (#99-601-CM, Corning). The isolation chamber was accessed for serial evaluation of graft function and viability on days 0, 7, 14, 21, 28, 45, 75, and 90.

### In vitro GSIS

To assess the ability of device-housed islets to secrete insulin in response to glucose exposure, perfusion-based GSIS in vitro assays were performed. Each glucose solution was continuously administered by syringe pump at a rate of 1.0 ml/min, starting with low glucose solution (2.5 mM glucose in modified Krebs-Ringer buffer) for 10 min, followed by high glucose solution (16.7 mM glucose in modified Krebs-Ringer buffer) for 30 min, followed low glucose plus arginine solution (2.5 mM glucose and 10 mM arginine) for 10 min. UF was collected for C-peptide measurement at the start of infusion, after 5 and 10 min of low glucose solution infusion, at 2, 3, 4, 5, 7, 10, 15, and 30 min poststart of high glucose infusion, and at 5 and 10 min poststart of low glucose plus arginine infusion.

### Rodent biocompatibility studies

Six 3-month old healthy male Lewis rats (299 to 333 g) (Charles River Laboratories) were used for initial in vivo implant test of the AD for biocompatibility, with size and shape of the AD and intended use scaled and implanted accordingly. Three or more animals are required to meet ISO 10993-6 testing for implant effects, *N* = 6 was selected balancing the well know safety profile of device materials but unknown reaction to device design. All rats were group-housed in a Midwest Critter Nation enclosure and were provided with shredded paper bedding and rotating autoclaved enrichment. Rats were provided ad libitum water and irradiated rodent diet food (Diet 2919 Teklad Global 19% Protein Rodent Diet, Harlan Laboratories) for the duration of the study. Room temperature was maintained at 20° to 27°C, humidity was maintained at 30 to 70%, and lights were programmed on a 12-hour on, 12-hour off light cycle. Rats were observed daily for general appearance, behavior, and body condition as a part of routine health monitoring and were weighed multiple times per week, and veterinary rounds were performed weekly.

Rats had a nose cone placed, and general anesthesia was administered with meloxicam subcutaneously (SC) (2 mg/kg) for pain management. Rats were positioned prone for a dorsal surgical site. The intended incision site was clipped of hair, and the site was prepped with chlorhexidine gluconate/isopropyl alcohol solution and draped with sterile towels. Incision was made between the shoulder blades and midback and with blunt dissection, and pockets were created bilaterally to accommodate the AD and the attached access port. The AD and attached access port were introduced into the subcutaneous pockets such that they were contralateral to each other. The incisions were closed in three layers using 5-0 absorbable monofilament suture and sealed with topical skin adhesive.

Rats received meloxicam SC (1 mg/kg) for 3 days for postoperative pain management. The incisions were monitored and allowed to heal for 7 days before port accessing. The port was aseptically prepped and accessed on days 7, 14, 21, 28, 35, 42, 49, 56, 63, 70, 77, 84, and 90, to assess disc patency. The composition of UF in the AD was evaluated on days 7, 14, 28, 56, and 90. Histological evaluation of the disc and surrounding tissue was performed at day 90 to address the presence and density of phagocytic cells, foreign body giant cells, the thickness and maturity of fibrous capsules and newly formed connective tissue surrounding the disc, and degeneration, necrosis, fatty infiltration, and foreign debris.

### Nonhuman primates

A total of seven NHPs, four Indian origin rhesus macaques (*Macaca mulatta*) (three male, one female) and three Mauritian origin cynomolgus macaques (*Macaca fascicularis*) (three male), were enrolled for device testing (table S5). All enrolled animals were healthy and confirmed tuberculosis negative and viral negative (macaque herpes B virus, simian retrovirus D, simian immunodeficiency virus, and simian T cell leukemia virus 1). Animals were aged 7.3 ± 1.1 years old and weighed 8.5 ± 1.0 kg. For this exploratory study, each individual animal was used to model a combination of conditions of interest, enrolling the two commonly used species of macaques used in transplantation modeling, these studies were not designed to achieve statistical significance or detect rare adverse events. Animals are presented individually for clarity, and where appropriate, grouping by similar experimental condition has been performed to evaluate trends and define expected variability for future modeling.

To realize the need for frequent blood draws while avoiding confounding effects from restraint, sedation, and pain, all animals were implanted with single-incision peripherally inserted vascular access ports as previously described (*101*). All animals were trained to cooperate with examination, blood collection, and general husbandry activities as part of the behavioral management program (*102*, *103*).

Animals were fed a standardized diet of either 2055C Certified Teklad Global 25% Protein Primate Diet or 7195 Teklad High Fiber Primate Diet (Envigo, Madison, WI, USA). A standardized enrichment program was used for the duration of the study, including fresh fruits and vegetables, grains, beans, and nuts, as well as a children’s multivitamin.

Animal behavior and clinical status were evaluated at least twice daily. Scheduled physical examinations per protocol and semiannual comprehensive veterinary examinations were performed on all animals. Animals were housed in same-sex pairs, except in rare cases of demonstrated social incompatibility, in which singly housed animals remained in close proximity with social conspecifics maintaining visual, auditory, and olfactory contact at all times. An environmental enrichment program including social play, toys, music, and regularly scheduled access to a large exercise and swimming area was provided to encourage sensory engagement, enhance foraging behavior and novelty seeking, promote mental stimulation, increase exploration, play, and activity levels, and strengthen social behaviors, increasing the proportion of time animals spent on species-typical behaviors. All animals enrolled in this study were offered equal access and time for exercise and identical enrichment activities.

### Surgical implantation of CS device

#### CS procedure

NHPs were sedated with ketamine intramuscularly (IM) (10 to 12 mg/kg) with or without midazolam IM (0.1 mg/kg) and received buprenorphine IM (0.03 mg/kg) and ketoprofen IM (1.0 mg/kg) for pain management. NHPs were intubated for general anesthesia and were positioned either lateral-prone or supine for either posterior flank or anterior abdominal surgical sites, respectively. The intended incision sites were clipped of hair, and the sites were widely prepped with chlorhexidine gluconate/isopropyl alcohol solution and draped with sterile towels. The incision sites were infiltrated with 1% lidocaine (1:5 dilution). Incision was made and with blunt dissection, a proximal pocket was created to accommodate the AD, and a 5- to 6-cm pocket was created medially to accommodate the pump and cell house components within the subcutaneous tissue. An additional subcutaneous pocket was dissected to accommodate the inductive charging coil for cooperative charging, and a distal pocket was created to accommodate the outflow from the device. Each component of the device was flushed with saline to ensure patency, and the device was assembled before placement within the subcutaneous pockets. The incisions were closed in four layers using 5-0 absorbable monofilament suture and sealed with topical skin adhesive.

#### CS-h procedure

In a similar manner to the CS procedure, NHPs were sedated with ketamine IM (10 to 12 mg/kg) with or without midazolam IM (0.1 mg/kg) and received buprenorphine IM (0.03 mg/kg) and ketoprofen IM (1.0 mg/kg) for pain management. NHPs were intubated for general anesthesia and positioned as dictated for the intended surgical site incisions. Sites were prepped and draped in a similar fashion receiving local anesthetic as described above at the intended incision sites. Incision was made and with blunt dissection, and a subcutaneous pocket proximal to the incision was created to accommodate the combined accumulation chamber/cell house component of the device. Similarly, blunt dissection was used to create a subcutaneous (approximately 8 cm by 0.5 cm) tunnel distal to the incision to accommodate the outflow port and tubing. The device components were flushed with saline to ensure patency, and the AD was introduced to the pocket with the tubing directed into the tunnel directly caudally. The incisions were closed in the same fashion as described above.

For both CS and CS-h devices, the NHPs were allowed to heal for at least 7 days before loading, allowing for sufficient UF accumulation. NHPs received a combination of buprenorphine IM (0.01 to 0.03 mg/kg) and/or either ketoprofen IM (1.0 mg/kg) or 100 mg of ibuprofen per os (PO) as needed for at least 3 days following the device implantation procedure. In the CS, the pump was controlled via Bluetooth.

### Diabetes induction

Diabetes was induced in four animals using pharmaceutical-grade STZ (streptozotocin, Zanosar, Sicor Pharmaceutics, Irvine, CA, USA) using methods previously described by this laboratory (*104*, *105*). After verifying appropriate hydration, a single dose of STZ (90 to 100 mg/kg) was infused intravenously. Diabetes was confirmed by persistent hyperglycemia (>300 mg/dl on at least two consecutive readings), the need for exogenous insulin to maintain target blood glucose (BG) levels, and the absence of C-peptide response to metabolic challenge. Diabetic NHPs were treated using glargine and lispro in combination on a sliding scale to target preprandial blood glucose levels between 50 and 150 mg/dl and to prevent the development of ketosis.

### Islet transplantation and biopsy

Animals were sedated using ketamine (8 to 12 mg/kg) with or without midazolam (0.1 mg/kg) for islet transplantation. Following aseptic preparation of the needle access site, a 19-gauge Huber needle was used to percutaneously access the cell house port. A syringe loaded with porcine islets (30,000 to 350,000 IEQ) was used to gently infuse the cell graft product, with or without hydrogel support. Supportive insulin was used as necessary in the posttransplant period for diabetic animals. Biopsies were performed longitudinally following islet loading to assess graft function and viability as well as to characterize the UF within the device.

### Laboratory testing

For complete blood counts, venous blood samples were collected into EDTA-treated microtainers and analyzed using the Advia 2120 hematology analyzer (Siemens Healthineers USA, Malvern, PA, USA). For chemistry panels, venous blood samples were collected into serum separator tubes and centrifuged to obtain serum. Chemistry panels were analyzed using an AU480 chemistry analyzer (Beckman Coulter, Brea, CA, USA).

### Graft assessment

#### Histological processing

Islet cells were fixed in 10% formalin, paraffin-embedded, and processed for routine histology. IHC was performed on islet biopsies taken from the devices as well as on the stroma surrounding the AD. Four-micrometer-thick sections of tissue were cut, and slides were loaded onto the Biocare Intellepath IHC staining instrument (Biocare Medical, Pacheco, CA, USA). Slides were deparaffinized through xylene and rehydrated through graded alcohol to water. If needed, heat retrieval was performed. Endogenous peroxide was quenched with 3% hydrogen peroxide followed by a protein serum block. Antibodies were applied followed by detection each for 30 min at room temperature (table S4). Slides were developed with 3,3'-diaminobenzidine (DAB) and counterstained with Mayer’s hematoxylin.

Following staining, biopsies were imaged using a Nikon Eclipse-800 M bright field/fluorescence/darkfield microscope equipped with a Nikon DXM1200 high-resolution digital camera and NIS Elements-D 5.02.00 Imaging software. The biocompatibility of both the device outflow and the AD were also assessed upon removal of the device.

#### Histological assessment

All biopsies from the CS devices were reviewed by a board-certified veterinary pathologist and scored according to table S5 to assess the degree of insulin immunoreactivity, infiltration of the cell product by inflammatory cells, islet fragmentation, and cell viability. The tissue-device interface was also assessed for device biocompatibility.

#### C-peptide

UF was collected and treated with bovine lung aprotinin (Millipore-Sigma, Darmstadt, Germany) at a ratio of a minimum of 500 kU to 1 ml of sample. C-peptide was measured via commercial Porcine C-Peptide ELISA (Mercodia, Uppsala, Sweden) (*106*).

#### Hemoglobin A1c

HbA1c was measured from whole blood using a point-of-care DCA Vantage Analyzer (Siemens Healthineers USA, Malvern, PA, USA).

#### Cytokines

Cytokines were measured in UF as well as serum using the NHP XL Cytokine Luminex Performance Premixed Kit (Bio-techne, MN, USA) (*107*).

#### IgG, IgM, and antidonor

Frozen donor peripheral blood leukocytes were thawed and, after washing with complete RPMI, resuspended at 4 × 10^6^ cells/ml in fluorescence-activated cell sorting (FACS) buffer [phosphate-buffered saline (PBS) containing 2% fetal bovine serum]. Fifty microliters of prepared donor cell suspension was aliquoted into each well of U-shape 96-well plate and mixed with 50 μl of recipient serum, incubated at room temperature for 30 min, and then the cells are washed three times in PBS. After the final centrifugation, cells are resuspended in 100 μl of FACS buffer containing fluorescein isothiocyanate–anti-IgG, phycoerythrin (PE)–anti-CD20, PE Cy7–anti-CD3, and LIVE/DEAD Fixable Aqua dye and incubated at room temperature for 20 min, and cells were washed twice and resuspended in FACS buffer. The stained cells were analyzed by a BD FACS Canto II flow cytometer. Detection of anti-IgG or anti-IgM levels on CD3^+^ or CD20^+^ gated cells represent the amount of donor-specific antibodies in each recipient’s serum.

#### Complement

Complement activation in UF was assessed using complement C4a as it is both highly stable persistent in serum. C4a was measured using a commercially available ELISA (Quidel, San Diego, CA).

### Data analysis

Values are reported as mean ± SEM unless otherwise specified. Statistical analysis and graphical representation of data were performed using Prism version 9.2.0 (GraphPad Software, San Diego, CA, USA). Kaplan-Meier time-to-event analysis was used to assess differences in graft survival, stratified by device, dose, and hydrogel. All histopathological scoring was performed by a board-certified veterinary pathologist with graft assessment including viability, islet fragmentation, insulin production, and inflammatory infiltration of cell product.
