# Supplementary material for: A bioengineered artificial interstitium supports long-term islet xenograft survival in nonhuman primates without immunosuppression
Source: Sci Adv. 2024 Jan 5;10(1):eadi4919. doi: 10.1126/sciadv.adi4919 (PMC10776017; doi:10.1126/sciadv.adi4919)
Supplement: Supplementary file 1 — Figs. S1 to S6 Tables S1 to S6 Legend for data S1 [file sciadv.adi4919_sm.pdf]

Science Advances
AAAS

Supplementary Materials for

A bioengineered artificial interstitium supports long-term islet xenograft survival in nonhuman primates without immunosuppression

Scott H. Oppler *et al.*

Corresponding author: Melanie L. Graham, graha066@umn.edu

Sci. Adv. **10**, eadi4919 (2024)
DOI: 10.1126/sciadv.adi4919

The PDF file includes:

Figs. S1 to S6
Tables S1 to S6
Legend for data S1

Other Supplementary Material for this manuscript includes the following:

Data S1

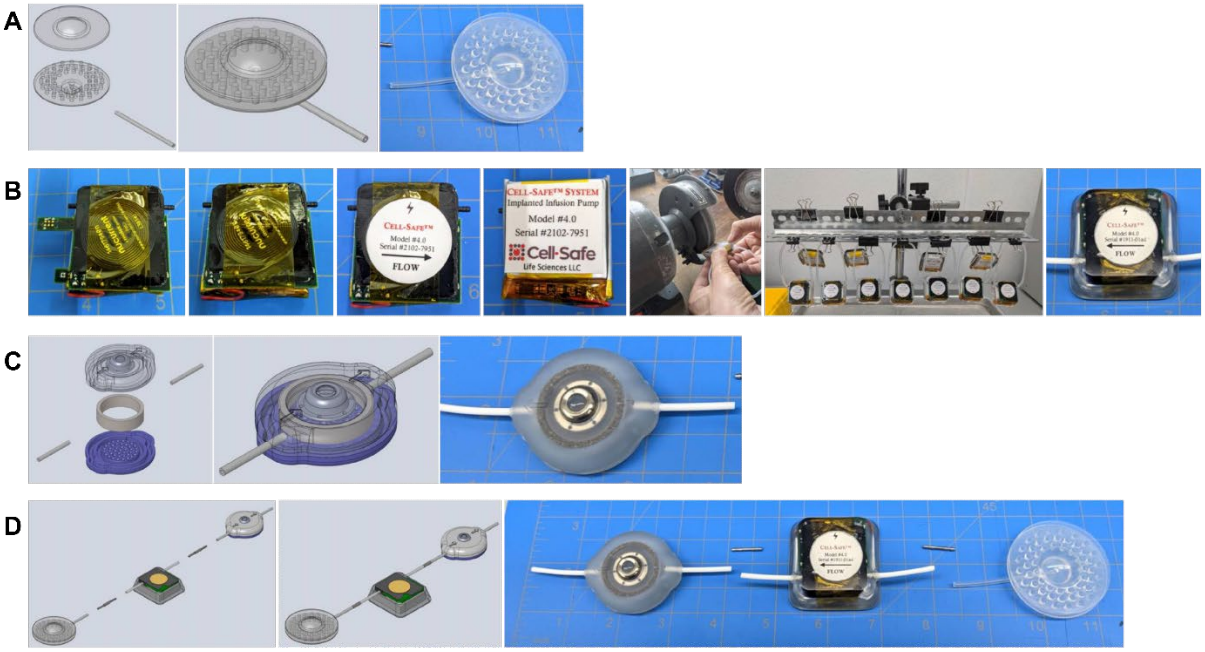

Fig. S1. CS device components and assembly. A) The accumulation chamber (left-right): exploded view, assembled accumulation chamber as modeled, and the assembled accumulation chamber. B) The piezoelectric pump (left-right): pump components assembled, pump tabs removed, top and bottom decals applied, grinding sharp edges after potting, dip coating the pumps, and completed pump unit. C) The cell house (left-right): exploded view, assembled cell-house as modeled, and assembled cell house. D) CS device components (left-right): exploded view, assembled CS device as modeled, and actual 3 component CS device.

Table S1. CS device media characteristics following islet loading *in vitro*. Data are presented as median ± IQR.

	d+0	*d+7*	*d+14*	*d+21*	*d+30*	*d+45*	*d+74*	*d+90*
pH	7.1±0.1	7.1±0.3	7.1±0.2	7.2±0.3	7.2±0.3	7.1±0.2	7.1±0.2	7.1±0.2
PCO2	9.9±1.7	8.2±5.1	8.2±4.6	8.4±1.5	8.0±1.6	8.6±1.5	8.3±2.1	9.3±1.1
PO2	150.0±28.0	137.0±35.0	145.0±32.0	144.5±27.0	138.5±44.0	146.5±25.0	145.0±33.0	147.0±30.0
Na	137.0±5.0	136.0±1.0	137.0±1.0	137.0±1.0	136.0±2.0	136.0±1.0	135.0±1.0	137.0±0.0
K	5.0±0.2	5.0±0.2	5.0±0.2	5.0±0.1	4.9±0.2	5.0±0.1	4.9±0.1	4.9±0.1

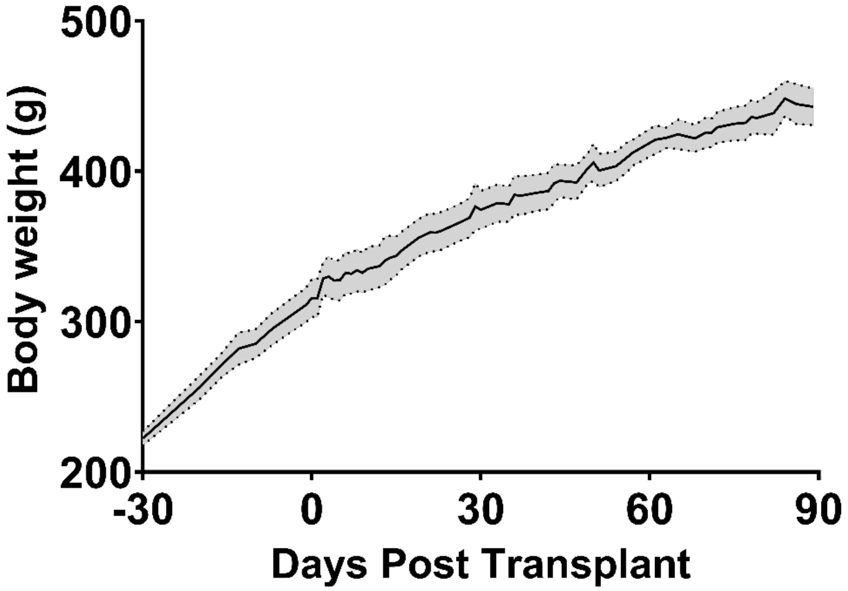

Fig. S2. Weight gain in rats implanted with the accumulation disc was appropriate to age.

Table S2. Ultrafiltrate characterization. Ions, low molecular weight molecules, and protein in ultrafiltrate generated by the accumulation disc implanted in rats without islet loading (n=6, mean ± SEM).

	d7	d14	d28	d56	d90	Human reference range
Urea Nitrogen (BUN) (mg/dL)	16.2±0.3	18.2±0.4	17.5±0.5	14.5±0.2	15.8±0.9	8-20
Creatinine (mg/dL)	0.2±0.0	0.2±0.0	0.2±0.0	0.2±0.0	0.2±0.0	0.5-1.2
Calcium (mg/dL)	8.3±0.1	8.5±0.1	8.1±0.0	8.1±0.0	8.0±0.1	9-10.5
Phosphorous (mg/dL)	6.9±0.2	6.8±0.1	7.1±0.1	6.1±0.1	5.7±0.1	3-4.5
Magnesium (mg/dL)	1.7±0.0	1.7±0.0	1.7±0.0	1.6±0.0	1.6±0.0	1.5-2.4
Total Protein (g/dL)	3.1±0.1	3.1±0.0	3.0±0.0	2.6±0.1	2.5±0.1	6-7.8
Albumin (g/dL)	1.7±0.0	1.8±0.0	1.6±0.0	1.6±0.0	1.6±0.1	3.5-5.4
Globulins (g/dL)	1.35±0.0	1.3±0.0	1.4±0.0	1.0±0.1	1.1±0.1	2.5-3.5
Sodium (mmol/L)	144±0.4	144±0.2	147±0.4	145±0.4	146±0.3	136-145
Chloride (mmol/L)	108±0.3	108±0.2	110±0.4	109±0.2	109±0.2	98-106
Potassium (mmol/L)	4.7±0.1	4.7±0.1	4.5±0.1	4.3±0.1	4.5±0.1	3.5-5
Bicarbonate (mmol/L)	25.3±0.6	23.6±0.8	22.1±1.0	27.2±1.1	26.2±1.4	23-29
Osmolarity (calc.)	287±0.9	289±0.4	292±1.1	289±1.1	291±0.5	275-295
Total Bilirubin (mg/dL)	1.1±0.1	0.9±0.1	1.6±0.3	1.0±0.4	0.8±0.6	0.3-1.2
Alkaline Phosphatase (U/L)	87.3±2.1	77.7±1.5	60.5±2.7	37.7±1.4	25.8±1.2	36-150
Aminotransferase, alanine (ALT) (U/L)	4.3±0.3	6.2±0.6	4.0±0.0	2.8±0.2	3.3±1.0	<35
Aminotransferase, aspartate (AST) (U/L)	94.7±2.5	98.5±5.4	71.5±2.4	47.0±4.3	47.5±13.9	<35
Creatine Kinase (CK) (U/L)	93.8±16.8	90.3±8.35	93.0±24.4	50.8±9.2	73.7±23.8	30-170
Glucose (mg/dL)	82.5±5.0	80.3±8.3	54.2±9.9	78.5±12.4	87.8±11.5	60-99
Cholesterol (mg/dL)	38.5±2.0	41.8±2.2	35.8±2.3	29.5±1.9	24.2±2.3	
Amylase (U/L)	194±12.0	189±6.7	149±4.8	95.7±5.9	79.8±7.6	<110
pH	7.3±0.02	7.3±0.03	7.2±0.02	7.3±0.03	7.4±0.03	7.35-7.45
PCO2 (mmHg)	55.2±2.1	58.9±1.9	61.1±1.8	52±2.0	49.6±2.7	35-45
PO2 (mmHg)	55.2±5.5	59.8±3.2	75.2±3.1	67±3.7	70.2±4.9	80-100

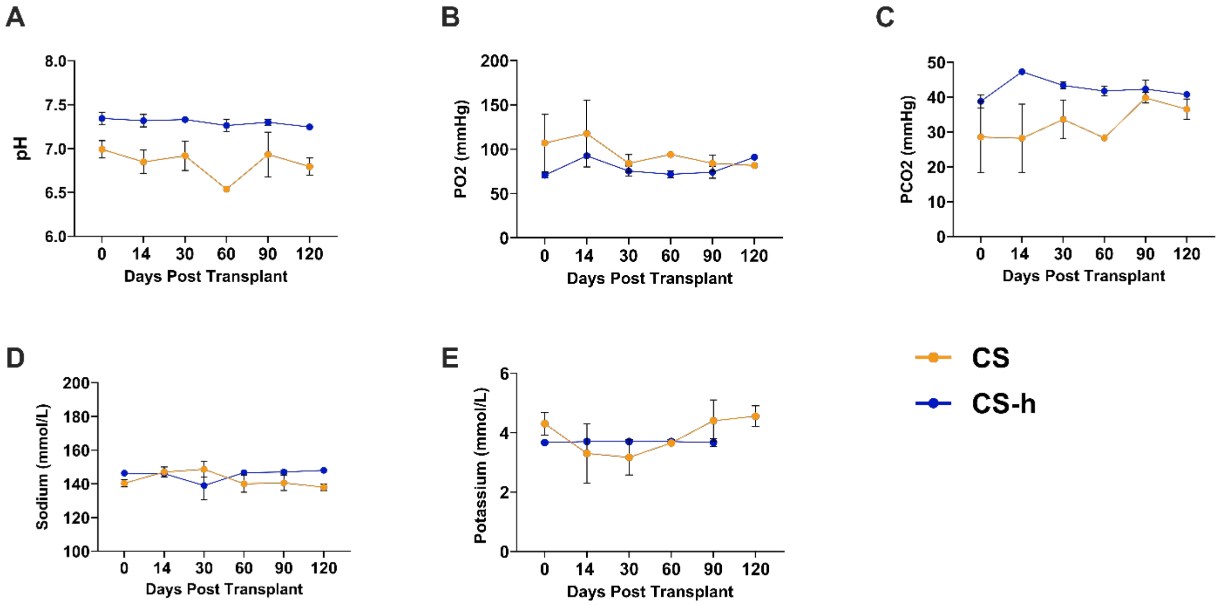

Fig. S3. Comparison of ultrafiltrate characteristics by device type in NHPs. Gasses and electrolytes in ultrafiltrate showed similar concentrations over time for both the CS device with an active pumping mechanism and the CS-h device which relies on diffusion.

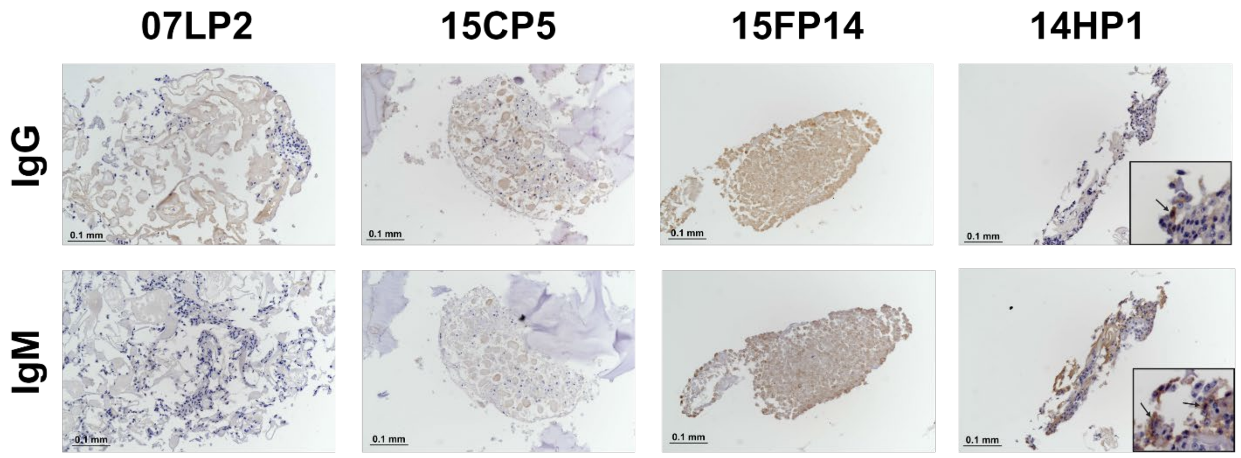

Fig. S4. Assessment of immune cell infiltration in islet biopsies by IgM, IgG staining. No cellular immunoreactivity was detected for IgG or IgM in 07LP2, 15CP5, and 15FP14. 14HP1 showed a single viable cell exhibiting moderate immunoreactivity for IgG and a few viable cells exhibiting moderate immunoreactivity for IgM (arrows within featured inset). Mild to moderate nonspecific background staining of cellular debris and matrix material is present in all specimens. Scale bar = 0.1 mm.

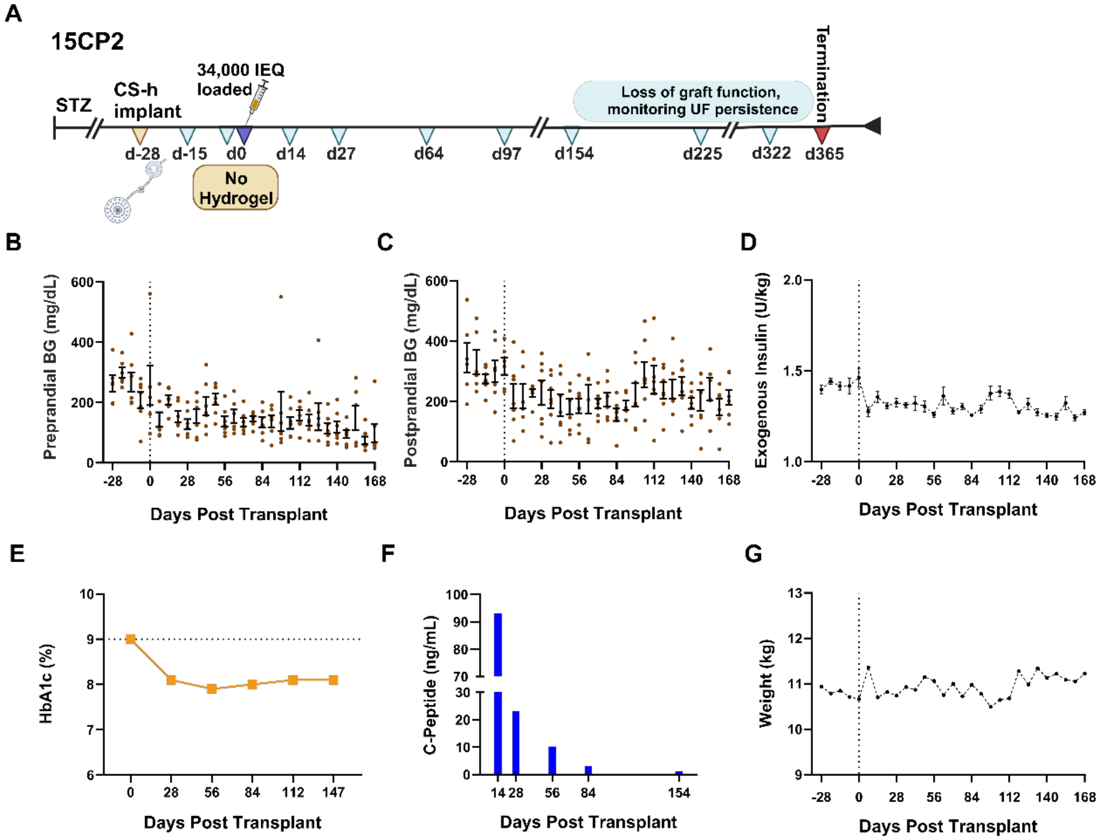

Fig. S5. Long-term metabolic effects of transplanted islets in the CS-h device in diabetic NHP 15CP2. A) Study timeline of 15CP2. Daily measures of B) preprandial and C) postprandial blood glucose (mg/dL) by day post transplant of 34,000 IEQ naked porcine islet cells into a CS-h device. D) Average daily exogenous insulin requirement (U/kg) by day post transplant. E) HbA1c (%) and F) porcine c-peptide (ng/mL) detected in device outflow by day post transplant. G) Weight by day post-transplant.

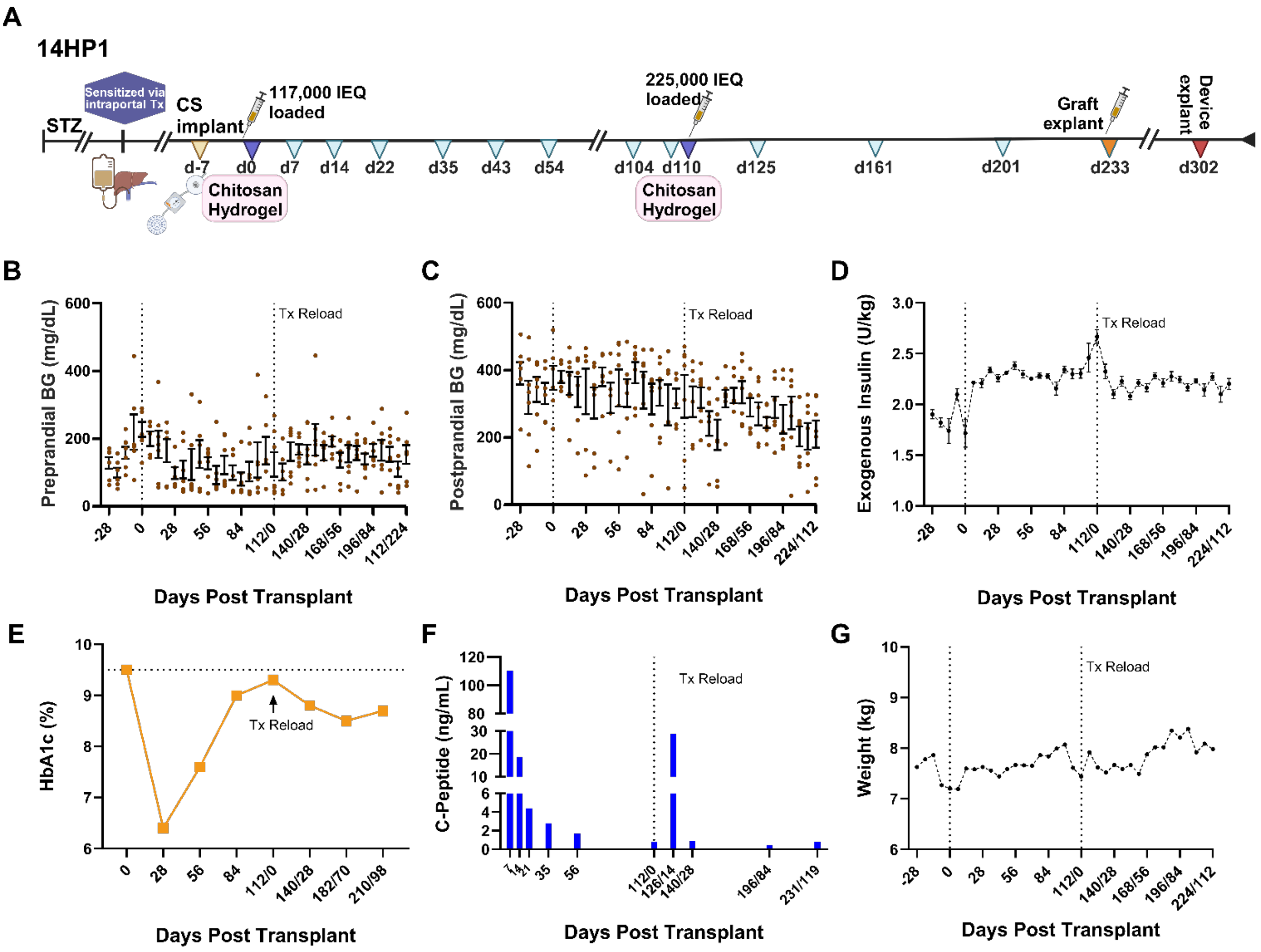

Fig. S6. Long-term metabolic effects of two loads of transplanted islets in the CS device in diabetic NHP 14HP1. A) Study timeline of 14HP1. Daily measures of B) preprandial and C) postprandial blood glucose (mg/dL) by day post transplant of 117,000 IEQ Islet-Mate supported porcine islet cells and then 225,000 IEQ Islet-Mate supported porcine islet cells into a CS device. D) Average daily exogenous insulin requirement (U/kg) by day post transplant. E) HbA1c (%) and F) porcine c-peptide (ng/mL) detected in device outflow by day post transplant. G) Weight by day post-transplant.

Table S3. NHP demographics, device assignments, and islet transplantation information.

<table>
<tr><th></th><th>ID</th><th>Macaque Species</th><th>Sex</th><th>Age (y)</th><th>Weight (kg)</th><th>Device</th><th>Hydrogel</th><th>Islet Dose (IEQ)</th><th>Islet Donor ID</th><th>Graft survival (days)</th><th>Graft disposition</th></tr>
<tr><td>Exploratory</td><td>07LP2</td><td>Rhesus</td><td>F</td><td>13.08</td><td>6.41</td><td>CS</td><td>Chitosan</td><td>130,000</td><td>P1096</td><td>>28</td><td>Planned termination</td></tr>
<tr><td rowspan="3">Non-diabetic</td><td rowspan="2">15FP14</td><td rowspan="2">Rhesus</td><td rowspan="2">M</td><td rowspan="2">5.76</td><td rowspan="2">14.16</td><td>CS</td><td>None</td><td>n/a</td><td>n/a</td><td>n/a</td><td>Device explant - infection</td></tr>
<tr><td>CS-h</td><td>None</td><td>34,000</td><td>P1147</td><td>225</td><td>Graft failure</td></tr>
<tr><td>18AP2</td><td>Cynomolgus</td><td>M</td><td>5.68</td><td>8.64</td><td>CS-h</td><td>None</td><td>92,000</td><td>P1169</td><td>15</td><td>Contaminated product</td></tr>
<tr><td rowspan="7">Diabetic</td><td rowspan="3">15CP2</td><td rowspan="3">Rhesus</td><td rowspan="3">M</td><td rowspan="3">5.20</td><td rowspan="3">7.63</td><td rowspan="2">CS</td><td rowspan="2">Chitosan</td><td>70,500</td><td>P1120</td><td>>7</td><td>Reload</td></tr>
<tr><td>125,000</td><td>P1122</td><td>>24</td><td>Contaminated product</td></tr>
<tr><td>CS-h</td><td>None</td><td>34,000</td><td>P1147</td><td>154</td><td>Graft failure</td></tr>
<tr><td>15CP5</td><td>Rhesus</td><td>M</td><td>7.83</td><td>8.6</td><td>CS</td><td>Capillary Alginate</td><td>334,901</td><td>P1132</td><td>182</td><td>Graft failure</td></tr>
<tr><td>14HP3</td><td>Cynomolgus</td><td>M</td><td>7.95</td><td>7.2</td><td>CS-h</td><td>None</td><td>343,000</td><td>P1169</td><td>56</td><td>Graft Failure</td></tr>
<tr><td rowspan="2">14HP1</td><td rowspan="2">Cynomolgus</td><td rowspan="2">M</td><td rowspan="2">5.56</td><td rowspan="2">6.96</td><td rowspan="2">CS</td><td rowspan="2">Chitosan</td><td>117,000</td><td>P1117</td><td>>110</td><td>Reload</td></tr>
<tr><td>225,000</td><td>P1126</td><td>>123</td><td>Planned graft removal</td></tr>
</table>

Table S4. IHC antibodies and staining methods.

Stain	Manufacturer	Cat #	Dilution	Antigen Retrieval	Detection	Chromogen
Insulin	Cell Signaling	4590S	1:100	None	Dako Rabbit Envision	DAB
Cleaved Caspase	Cell Signaling	9661	1:200	EDTA	Dako Rabbit Envision	DAB
Glucagon	Novus Biological	LS-C15055	1:200	None	Dako Rabbit Envision	DAB
Proinsulin	DHSB	GS-9A8	1:200	Citrate	Dako Mouse Envision	DAB
Factor VIII	Dako	A00082	1:400	Proteinase K	Dako Rabbit Envision	DAB
CD68	Dako	M0814	1:1000	Citrate	Dako Mouse Envision	DAB
CD163	FabGen	CD163-101-AP	1:1000	Citrate	Dako Rabbit Envision	AEC

Table S5. Islet graft biopsy scoring schema.

Assessment	Score	Interpretation
Biocompatibility	0	No tissue on slide
	1	Well organized granulation tissue (dense, lamellar connective tissue) with good vascularity (moderate to large numbers of small-caliber blood vessels) and few, scattered inflammatory infiltrates (primarily macrophages/siderophages)
	2	Poorly organized granulation tissue (more fibrin or disorganized collagen) with poor vascularity (no or small numbers of blood vessels) and large numbers of inflammatory infiltrates (primarily neutrophils) or large areas of necrosis
Viability of cell product	0	No islet cells/material present or too much artifact to interpret
	1	<25% of islet cells appear viable
	2	25-50% of islet cells appear viable
	3	50-75% of islet cells appear viable
	4	>75% of islet cells appear viable
Islet Fragmentation	0	No islet cells/material present or too much artifact to interpret
	1	Small number of islets exhibit fragmentation
	2	Moderate number of islets exhibit fragmentation
	3	Large number of islets exhibit fragmentation
Infiltration of Inflammatory Cells	0	No inflammatory cells present
	1	Few scattered inflammatory cells present
	2	Moderate #'s of inflammatory cells present
	3	Large numbers of inflammatory cells present
Insulin	0	No insulin staining present/No cells on slide to examine
	1	<25% of islet cells stain for insulin
	2	25-50% of islet cells stain for insulin
	3	50 -75% of islet cells stain for insulin
	4	>75% of islet cells stain for insulin

Table S6. Islet graft biopsy assessment.

Days post islet transplant (±5 days)		d+14	d+30	d+60	d+90	d+150	d+182	d+207
07LP2	Islet Fragmentation	3	3					
	Insulin Staining	4	3					
	Infiltration of inflammatory cells	0	0					
15FP14	Islet Fragmentation	3	3	3	2	3		
	Insulin Staining	4	4	4	4	4		
	Infiltration of inflammatory cells	0	0	0	0	0		
15CP5	Islet Fragmentation	1	3	0	3	3	3	3
	Insulin Staining	3	4	4	4	4	4	4
	Infiltration of inflammatory cells	0	0	0	0	0	0	0

Data S1. Raw data for all processed data presented in the manuscript.
